# Sex differences in the association between fatty liver index and biological aging: A mediation analysis of insulin resistance in a cross-sectional study

**DOI:** 10.1097/MD.0000000000046152

**Published:** 2026-05-12

**Authors:** Jia Yang, Haifeng Liu, Weimin Zhao, Tiejun Liu

**Affiliations:** a College of Chinese Medicine, Changchun University of Chinese Medicine, Changchun City, Jilin Province, China; b Department of Neurology, First Affiliated Hospital to Changchun University of Chinese Medicine, Changchun City, Jilin Province, China; c Department of Gastroenterology, First Affiliated Hospital to Changchun University of Chinese Medicine, Changchun City, Jilin Province, China.

**Keywords:** aging, cross-sectional, fatty liver, insulin resistance, NHANES, sex differences

## Abstract

Aging challenges global healthcare systems, with fatty livers potentially relevant to the process. This study sought to investigate the sex-specific associations of fatty liver index (FLI) with biological aging (BA), assessed via phenotypic age (PhenoAge) and PhenoAge acceleration (PhenoAgeAccel), and explored the mediating role of the metabolic score for insulin resistance (METS-IR). Utilizing nationally representative data from 16,479 American adults in the National Health and Nutrition Examination Survey (NHANES 1999–2020), this cross-sectional study employed weighted multivariable regression, receiver operating characteristic curves, restricted cubic splines, threshold effect analyses, sensitivity analyses, and mediation analyses. FLI was positively correlated with BA overall (*P < *.001), with significant sex interaction effects observed (*P*_interaction_ < .05). For every 1 − standard deviation increase in FLI, females exhibited stronger associations with PhenoAge (β_female_ = 3.33, 95% confidence interval [CI]: 3.07–3.58; β_male_ = 2.54, 95% CI: 2.20–2.89) and PhenoAgeAccel risk (OR_female_ = 1.89, 95% CI: 1.76–2.03; OR_male_ = 1.57, 95% CI: 1.46–1.69; all *P < *.001). The area under the curve of FLI for PhenoAgeAccel was higher in females (0.73, 95% CI: 0.72–0.75) than in males (0.67, 95% CI: 0.66–0.68). Restricted cubic splines revealed a biphasic upward trend in the FLI-BA relationship across both sexes (*P*_nonlinear_ < .001), with sex-divergent threshold effects (*P*_likelihood ratio_ < .001). METS-IR partially mediated the FLI-BA association, with a greater percentage of mediation effect in males (PhenoAge: 20.23%; PhenoAgeAccel: 36.84%) than females (8.73%; 15.93%). A robust positive association exists between FLI and BA, partially mediated by METS-IR. Females exhibited stronger association strength, while males demonstrated a greater proportion of the METS-IR’s mediation effect.

## 1. Introduction

Global population aging, characterized by rising life expectancy and declining birth rates, presents a significant challenge to healthcare systems worldwide.^[1]^ Epidemiological projections indicate individuals aged ≥60 years will constitute 16% of the global population by 2030, rising to 22% (2.1 billion) by 2050.^[2]^ In response to this accelerating demographic shift, the World Health Organization has designated 2021 to 2030 as the UN Decade of Healthy Aging.^[3]^ Accelerated aging is a well-established determinant of multiple chronic disorders, creating substantial healthcare burdens and contributing significantly to age-related mortality, thereby necessitating strategies to quantify and mitigate aging processes.^[4]^ Biological age, a metric of physiological decline, surpasses chronological age (CA) in predicting health deterioration.^[5]^

Phenotypic age (PhenoAge) as a validated biological age estimation method, using clinical biomarkers via Gompertz mortality modeling, provides effective discrimination of mortality risk among individuals with identical CA.^[6]^ Its residual-based metric, PhenoAge acceleration (PhenoAgeAccel), predicts excess mortality when positive.^[7]^ Widely employed as an endpoint, PhenoAge quantifies aging acceleration induced by environmental exposures, health behaviors, and interventions, establishing PhenoAge and PhenoAgeAccel as robust biological aging (BA) biomarkers strongly associated with adverse health outcomes.^[8–10]^

Metabolic dysfunction-associated steatotic liver disease (MASLD) provides a new framework for the categorization of steatotic liver disease by emphasizing the interplay among hepatic steatosis and cardiometabolic dysfunction, thereby superseding the term nonalcoholic fatty liver disease. This revised nosology utilizes multi-omics strategies to achieve a stratification with greater precision than the previous criteria for metabolic dysfunction-associated fatty liver disease.^[11]^ MASLD fundamentally correlates with metabolic well-being and now represents the most widespread chronic liver disorder worldwide. Studies report metabolic syndrome prevalence rates reaching 65.3% in diabetes mellitus (DM) patients, with a male predominance of 51.41%. This figure is particularly pronounced in Eastern European and Middle Eastern populations, where the prevalence exceeds 70%. Projections estimate that by 2045, the number of incident cases will reach 667.58 million.^[12,13]^

The fatty liver index (FLI) operates as a pivotal indicator denoting disease progression and severity in MASLD, demonstrating considerable associations with multiple aging-related disorders.^[14]^ A nationwide Korean cohort of >1 million middle-aged adults revealed that FLI ≥ 60 was correlated with a 23% rise in DM risk, while a German cohort (n = 2920) linked elevated FLI to a 28% rise in chronic kidney disease (CKD) risk.^[15,16]^ Similar correlations extend to hypertension (HTN), depression, and sleep disorders.^[17,18]^ Mechanistically, insulin resistance (IR) is critically linked to MASLD, potentially driving these geriatric conditions through metabolic dysregulation, chronic inflammation, oxidative stress, and vascular dysfunction.^[19,20]^ Nevertheless, the relationship between FLI and BA remains unestablished, and IR’s potential mediating role in this association is poorly characterized. Notably, FLI exhibits pronounced sexual dimorphism: its MASLD detection threshold is 50% lower in females, with sex-specific patterns also observed in FLI-cognitive dysfunction and FLI-cardiovascular disease (CVD) relationships.^[14]^ For instance, Higashiura et al demonstrated that HTN risk trajectories showed linear progression in males versus rapid initial then plateaued increases in females across ascending FLI strata.^[21]^ However, whether such sex disparities extend to FLI-BA association and IR’s mediating effects requires investigation. Addressing these knowledge gaps may facilitate the development of sex-stratified management strategies for both MASLD and the aging process.

The current project quantified associations of FLI and BA (assessed via PhenoAge and PhenoAgeAccel) in a nationally representative American cohort. This research was facilitated by the National Health and Nutrition Examination Survey (NHANES) from 1999 to 2020. We hypothesized that: FLI exhibits a positive association with BA, this relationship is mediated by IR, and sex differences exist in both the FLI-BA association and the proportion of IR mediation effect. The following analytical strategy was implemented. First, comprehensive characterization of baseline demographics, lifestyle factors, and comorbidities that may confound the association of FLI with BA was conducted. Second, assessments of linear and nonlinear FLI-BA associations were performed. Third, evaluation of the robustness of the findings was carried out through progressively adjusted multivariable models and sensitivity analyses. Finally, the quantification of the mediation effects of IR was undertaken. Sex-stratified analyses were systematically integrated throughout all the stages. Collectively, this research advances understanding of relationships between FLI and BA, thus providing insights that could inform sex-specific public health strategies targeting MASLD and aging trajectories.

## 2. Materials and methods

### 2.1. Study population

**Figure 1.**
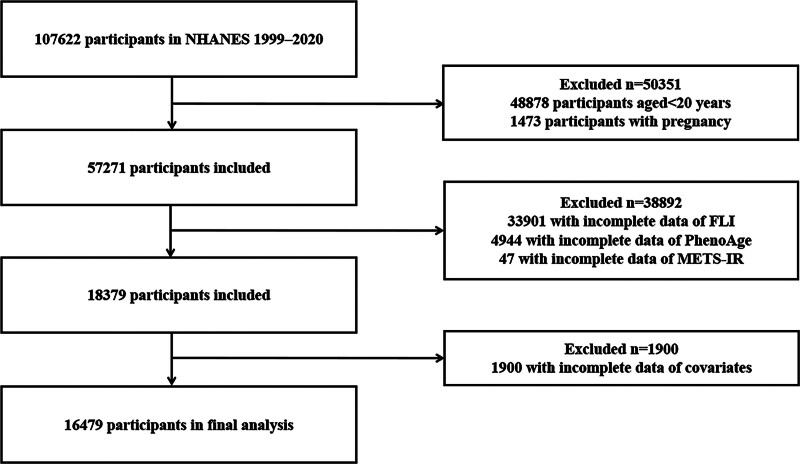
Flow chart. FLI = fatty liver index, METS-IR = metabolic score for insulin resistance, NHANES = National Health and Nutrition Examination Survey, PhenoAge = phenotypic age.

De-identified information collected from 11 NHANES survey cycles (1999–2020), obtained through standard protocols, and was fully utilized in this cross-sectional analysis. To obtain nationally representative data, a multistage probability sampling approach was utilized, encompassing interviews, physical examinations, and mobile laboratory assessments. The study protocol was approved by the National Center for Health Statistics Research Ethics Review Board, and written informed consent was obtained from all participants prior to data collection (https://www.cdc.gov/nchs/nhanes/about/erb.html). As this secondary analysis used only aggregated and deidentified data without any protected health information, no additional ethical approval was required. A sequence of exclusions was applied (Fig. 1). First, participants <20 years of age (n = 48,878) and pregnancies (n = 1473) were removed. Next, subjects who lacked complete data for FLI (n = 33,901), PhenoAge (n = 4944), and the metabolic score for IR (metabolic score for insulin resistance [METS-IR]; n = 47) were excluded. Finally, those with incomplete covariates (n = 1900) were removed, resulting in a final cohort of 16,479 adults.

### 2.2. Assessments of FLI and IR

FLI utilized basic clinical and laboratory indicators including triglycerides (TG), body mass index (BMI), γ-glutamyl transferase, and waist circumference. According to the FLI assessment model,^[22]^ scores ranged from 0 to 100 and were calculated as:


$$\begin{array}{l} FLI=\frac{e^{0.953\;\;\times \;ln\left[TG\;\left(mmol/L\right)\right]\;+\;0.139\;\times \;BMI\;+\;0.718\;\;\times \;\;ln[GGT\left(U/L\right)\;]\;\;+\;0.053\;\times \;WC\;\left(cm\right)\;-15.745}}{1+e^{0.953\;\times \;ln\left[TG\;\left(mmol/L\right)\right]\;+\;0.139\;\times \;BMI\;+\;0.718\;\;\times \;\;ln[GGT\left(U/L\right)\;]\;\;+\;0.053\;\times \;WC\;\left(cm\right)\;-15.745}}\times 100 \end{array}$$

IR was evaluated using METS-IR, which was derived from fasting plasma glucose, TG, BMI, and high-density lipoprotein levels.^[23]^ This biochemical index served as a clinically reliable surrogate marker for quantifying IR, and was computed as:


$$\begin{array}{l} METS\text{-}IR=\frac{\ln\left[2\times FPG\;\;\;\left(mg/dL\right)+TG\;\left(mg/dL\right)\right]\times BMI\;\left(kg/m^{2}\right)}{\ln HDL\left(mg/dL\right)} \end{array}$$

### 2.3. Assessment of BA

BA was quantified using PhenoAge and PhenoAgeAccel metrics. The PhenoAge algorithm was a validated multivariable predictor of mortality risk, which integrated CA, albumin, creatinine, and 6 clinical biomarkers to estimate biological age.^[24]^ The computational details were provided in Table S1, Supplemental Digital Content, https://links.lww.com/MD/Q758. PhenoAgeAccel was computed as the arithmetic difference between PhenoAge and CA. Positive residuals, defined as values greater than CA, were indicative of accelerated BA. Conversely, nonpositive values suggested BA at or below chronological expectations.^[25]^ Finally, this continuous measure was dichotomized for categorical risk assessment in subsequent analyses.

### 2.4. Covariates

To account for potential confounders, established demographic, lifestyle, and clinical covariates were analyzed. The demographic adjustment included age, sex, race, education, marital status, and the poverty-income ratio (PIR < 1, denoting below-poverty status).^[26]^ The lifestyle factors involved smoking status, drinking status, and physical activity. The measurement of physical activity was quantified as the metabolic equivalent of task minutes per week, and categorized as low (<500) or high (≥500).^[27]^ Clinically relevant covariates, including HTN, DM, CVD, CKD, and cancer, were analyzed as covariates, with detailed diagnostic criteria provided in Table S2, Supplemental Digital Content, https://links.lww.com/MD/Q758.

### 2.5. Statistical analysis

The analysis took into account the intricate survey design of NHANES by using the “survey” package in R to incorporate clustering, stratification variables, and individual sampling weights to generate nationally representative estimates. Baseline characteristics were stratified by sex. An assessment of continuous variables’ normality was conducted through the implementation of Kolmogorov–Smirnov tests. The analysis of typically-distributed data was conducted through the implementation of weighted *t*-tests, with the resulting data set expressed as the mean ± standard error. In contrast, cases involving non-normally distributed data were subjected to weighted Mann–Whitney tests, with the results expressed as median values (first quartile, third quartile). Weighted χ² tests were used to compare categorical variables, and the results were reported as unweighted counts and weighted proportions.

Multivariable linear/logistic regression analyses evaluated the associations of FLI with BA (PhenoAge and PhenoAgeAccel) across 3 cohorts: the whole population, males, and females. We employed 3 progressive adjustment models. Model 1: unadjusted. Model 2: demographic-adjusted. Model 3: further adjusted for lifestyle behaviors and clinical covariates. Multiplicative interaction terms were used to test for effect modification by sex. Variance inflation factors <5 in model 3 confirmed the absence of multicollinearity. The FLI was analyzed in 2 distinct ways: first as a continuous variable, and then as a categorical variable divided into quartiles.

Restricted cubic spline (RCS) curves with 4 optimal nodes were used for the purpose of assessing potentially nonlinear associations of FLI with BA, balancing flexibility with parsimony. The evaluation of threshold effects was conducted through the implementation of 2-piecewise linear regression models, with likelihood ratio tests used to compare piecewise and linear fits and identify inflection points. All analyses were adjusted for model 3 covariates.

A series of sensitivity analyses underwent implementation to assess the reliability of the study’s findings: further adjustment for depression and sleep disorders in model 3, given the established links between these conditions and metabolic dysfunction and accelerated aging.^[28–31]^ Replication in unweighted models. Exclusion of participants aged ≥80 years to minimize potential confounding by advanced age. Concordance between primary and sensitivity estimates reinforced these results, thereby validating the findings.

Mediation analyses were performed for the purpose of evaluating the proportion of the FLI-BA association mediated by METS-IR in all 3 cohorts. To address methodological concerns from component overlap (BMI and TG in both FLI and METS-IR), we retained these variables as covariates after confirming multicollinearity was acceptable (all variance inflation factors <5). Final models of mediation analyses underwent additional adjustment with BMI and serum TG, along with model 3 covariates. Effects were decomposed into direct, indirect, and total components. Significance was assessed via nonparametric bootstrapping (1000 iterations; random seed = 1234). Effect sizes and 95% confidence intervals (CIs) were derived from bootstrap distributions.

All analyses were conducted using R (v4.3.1), and computational efficiency was enhanced by the *Z*stats 1.0 platform (http://www.zstats.net) for automated table generation and visualization. Statistical significance was established to indicate a *P*-value < .05 (2-tailed) for the observed data.

## 3. Results

### 3.1. Baseline characteristics

Data from 16,479 adults (8264 men and 8215 women) who participated in NHANES between 1999 and 2020 were analyzed in this cross-sectional study. Sex-stratified baseline characteristics are presented in Table 1. Males exhibited significantly higher FLI (60.32 vs 39.64), METS-IR (42.49 vs 39.22), older PhenoAge (56.93 vs 55.67 years), and greater PhenoAgeAccel risk (49.04% vs 34.63%; all *P* < .001) than females. Pronounced sex disparities were observed across most covariates. Females exhibited significantly higher levels of high-density lipoprotein, C-reactive protein, and lymphocyte percentage (all *P* < .01). There were greater proportions of females in older age groups, divorced/separated/widowed marital status, higher educational attainment, poverty prevalence, and in the prevalence of cancer, CKD, depression, and sleep disorders (all *P* < .01). Males exhibited significantly higher levels of TG, BMI, γ-glutamyl transferase, fasting plasma glucose, albumin, creatinine, mean cell volume, and alkaline phosphatase (all *P* < .05). Additionally, the prevalence of Mexican ethnicity, smoking, alcohol consumption, engagement in high physical activity, as well as the incidence rates of DM, CVD, and MASLD were remarkably elevated in this group (all *P* < .001). The incidence of HTN and the leukocyte count were comparable between sexes (both *P* > .05).

**Table 1:** Participant baseline characteristics.

Variable	Whole (n = 16,479)	Males (n = 8264)	Females (n = 8215)	*P*
Age (yr)	46.00 (34.00, 59.00)	46.00 (33.00, 58.00)	47.00 (34.00, 60.00)	<.001
Race, n (%)				<.001
Mexican American	2993 (7.81)	1529 (8.74)	1464 (6.89)	
Non-Hispanic White	7605 (70.08)	3879 (70.12)	3726 (70.03)	
Non-Hispanic Black	3279 (10.21)	1586 (9.29)	1693 (11.10)	
Other race	2602 (11.91)	1270 (11.84)	1332 (11.98)	
Marital status, n (%)				<.001
Never married	2107 (12.61)	1127 (14.17)	980 (11.09)	
Divorced/separated/widowed	4175 (21.95)	1527 (16.49)	2648 (27.27)	
Married/living with partner	10,197 (65.44)	5610 (69.34)	4587 (61.64)	
Education, n (%)				.003
<High school	4327 (16.34)	2298 (17.31)	2029 (15.38)	
High school	3864 (24.83)	1969 (25.24)	1895 (24.42)	
>High school	8288 (58.84)	3997 (57.45)	4291 (60.19)	
Poverty, n (%)	3062 (12.35)	1387 (10.86)	1675 (13.81)	<.001
Smoking, n (%)	7799 (47.75)	4658 (54.26)	3141 (41.41)	<.001
Alcohol intake, n (%)	11,611 (74.20)	6660 (82.16)	4951 (66.43)	<.001
Physical activity, n (%)				<.001
Low	6141 (33.02)	2643 (27.46)	3498 (38.44)	
High	10,338 (66.98)	5621 (72.54)	4717 (61.56)	
Cancer, n (%)	1544 (9.33)	726 (7.86)	818 (10.76)	<.001
Hypertension, n (%)	7009 (36.75)	3509 (37.12)	3500 (36.40)	.444
Diabetes mellitus, n (%)	3166 (14.21)	1671 (15.08)	1495 (13.36)	.008
Cardiovascular disease, n (%)	1841 (8.58)	1080 (9.57)	761 (7.61)	<.001
Chronic kidney disease, n (%)	2854 (12.53)	1366 (10.99)	1488 (14.04)	<.001
Depression, n (%)	1358 (7.16)	566 (5.98)	792 (8.31)	<.001
Sleep disorders, n (%)	2573 (15.14)	1184 (13.97)	1389 (16.28)	.004
MASLD, n (%)	1341 (42.19)	712 (46.23)	629 (38.27)	<.001
Body mass index (kg/m^2^)	27.64 (24.09, 32.10)	27.80 (24.70, 31.50)	27.40 (23.50, 32.88)	.032
Triglycerides (mmol/L)	1.20 (0.82, 1.76)	1.29 (0.88, 1.91)	1.11 (0.77, 1.64)	<.001
γ-Glutamyl transferase (U/L)	20.00 (14.00, 30.00)	24.00 (17.00, 37.00)	16.00 (12.00, 24.00)	<.001
Fasting plasma glucose (mg/dL)	99.00 (92.00, 108.00)	101.00 (94.20, 110.00)	97.00 (90.00, 105.00)	<.001
High-density lipoprotein (mg/dL)	51.00 (42.00, 62.00)	46.00 (39.00, 56.00)	57.00 (47.00, 68.00)	<.001
Albumin (g/dL)	4.20 (4.00, 4.50)	4.30 (4.10, 4.50)	4.20 (3.90, 4.40)	<.001
Creatinine, urine (mg/dL)	119.00 (71.00, 172.00)	139.00 (91.00, 194.00)	99.00 (57.00, 149.00)	<.001
C-reactive protein (mg/dL)	0.18 (0.08, 0.42)	0.15 (0.07, 0.33)	0.22 (0.09, 0.53)	<.001
Lymphocyte (%)	29.80 (24.60, 35.10)	29.50 (24.40, 34.80)	30.20 (24.80, 35.50)	<.001
Mean cell volume (fL)	90.10 (87.10, 92.90)	90.20 (87.40, 93.10)	89.90 (86.60, 92.90)	<.001
Alkaline phosphatase (U/L)	67.00 (55.00, 81.00)	68.00 (56.00, 81.00)	65.00 (53.00, 81.00)	<.001
Leukocyte count (1000 cells/μL)	6.40 (5.40, 7.80)	6.50 (5.50, 7.80)	6.40 (5.40, 7.80)	.355
Fatty liver index	50.78 (18.94, 82.43)	60.32 (29.06, 85.05)	39.64 (12.71, 78.23)	<.001
Metabolic score for insulin resistance	41.05 (33.78, 49.69)	42.49 (35.99, 50.31)	39.22 (31.88, 48.95)	<.001
Phenotypic age (yr)	56.41 (43.51, 69.77)	56.93 (44.05, 71.07)	55.67 (42.71, 68.69)	<.001
Phenotypic age acceleration, n (%)	7456 (41.74)	4309 (49.04)	3147 (34.63)	<.001

All continuous variables demonstrated non-normal distributions as assessed by the Kolmogorov–Smirnov test. Consequently, comparisons between the groups utilized a weighted Mann–Whitney test, with results expressed as median values (first quartile, third quartile). Categorical variables were compared through weighted chi-square tests and expressed as counts (unweighted) as well as proportions (weighted). The diagnosis of MASLD required both: hepatic steatosis, defined as a controlled attenuation parameter value ≥ 274 dB/m measured by vibration-controlled transient elastography; and the presence of at least one of the following cardiometabolic risk factors: body mass index ≥ 25 kg/m^2^ or waist circumference > 94 cm (for men), 80 cm (for women); fasting plasma glucose ≥ 5.6 mmol/L or 2-h post-load glucose ≥ 7.8 mmol/L or HbA1c ≥ 5.7% or type 2 diabetes or treatment for type 2 diabetes; blood pressure ≥ 130/85 mm Hg or specific antihypertensive drug treatment; triglycerides ≥ 1.70 mmol/L or lipid lowering treatment; high-density lipoprotein ≤ 40 mg/dL for men or ≤50 mg/dL for women or lipid lowering treatment. Individuals with viral hepatitis or excessive alcohol intake (>140 g/wk for women; >210 g/wk for men) were excluded. Due to the availability of controlled attenuation parameter measurements only in the 2017 to 2020 cycle of this study, the presentation of MASLD in this table is restricted to that period.

MASLD = metabolic dysfunction-associated steatotic liver disease.

**Figure 2.**
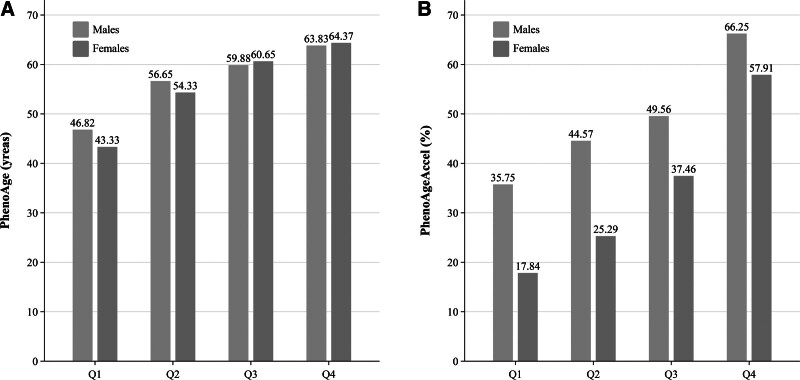
Sex-specific associations of fatty liver index quartiles with biological aging. Both sexes exhibited progressively elevated phenotypic age (PhenoAge), expressed by the median (A), and the proportions of phenotypic age acceleration [PhenoAgeAccel] (B) across ascending fatty liver index quartiles (Q1–Q4). PhenoAge = phenotypic age, PhenoAgeAccel = phenotypic age acceleration, Q = quartile.

As shown in Figure 2, both sexes presented progressively elevated PhenoAge and the proportions of PhenoAgeAccel (*P* < .001) across ascending FLI quartiles (Q1–Q4). See Table S3, Supplemental Digital Content, https://links.lww.com/MD/Q758 for details.

### 3.2. Association between FLI and BA

#### 3.2.1. Multivariable regression analysis

**Figure 3.**
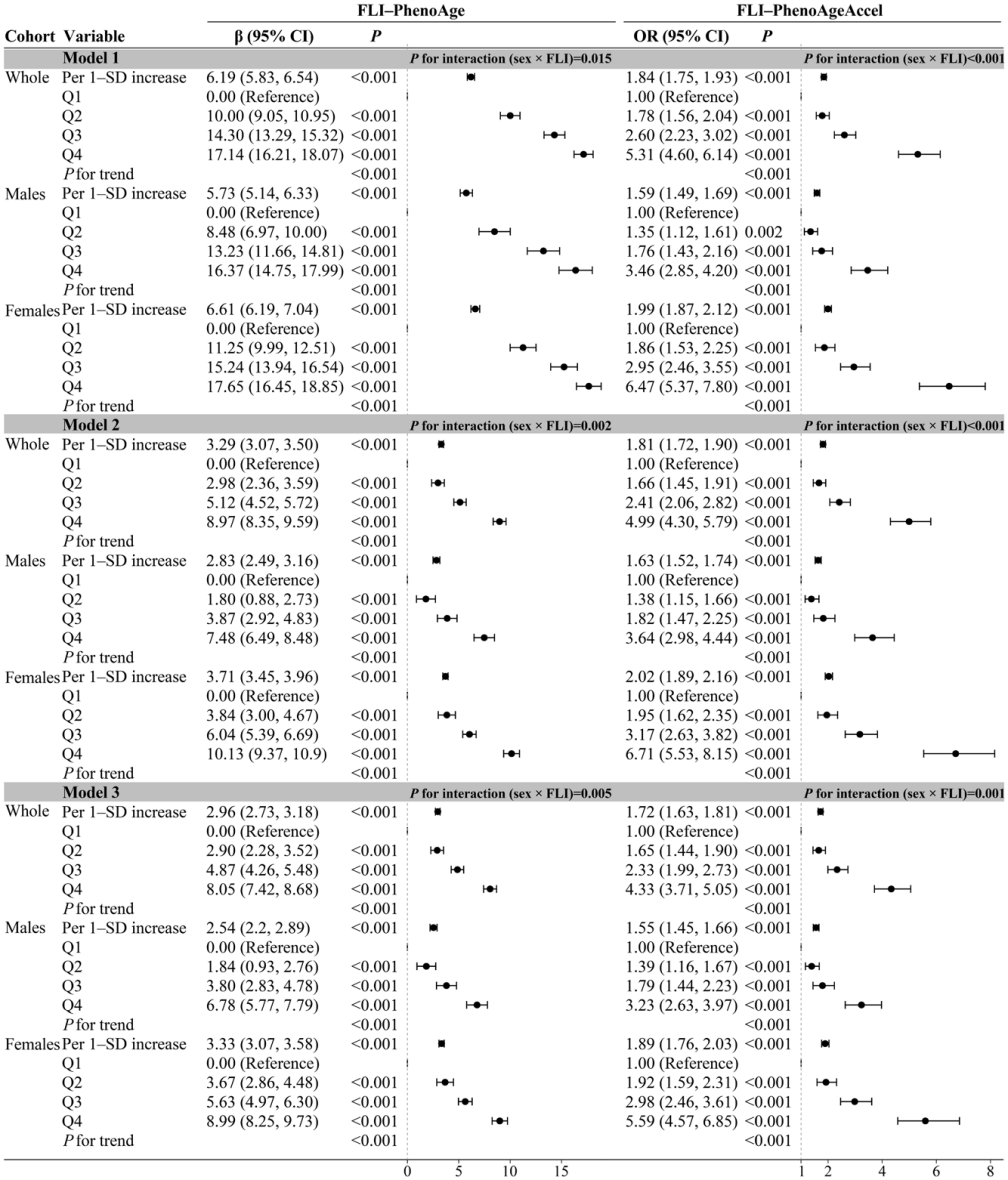
Multivariable regression analyses. Multivariate regression analyses for the associations of fatty liver index (FLI) with both phenotypic age (PhenoAge) and phenotypic age acceleration (PhenoAgeAccel) risk across whole cohort and sex-stratified groups. Model 1: unadjusted. Model 2: demographic adjusted. Model 3: further adjusted for lifestyle behaviors and clinical covariates. CI = confidence interval, FLI = fatty liver index, OR = odds ratio, PhenoAge = phenotypic age, PhenoAgeAccel = phenotypic age acceleration, Q = quartile, SD = standard deviation.

Multivariable linear and logistic regression analyses across all 3 models (Fig. 3) consistently demonstrated significant positive associations between FLI and BA (PhenoAge and PhenoAgeAccel) in the whole cohort and sex-stratified groups. In fully adjusted models (model 3) for the whole population, each standard deviation (SD) increases in FLI corresponded to a 2.96-year elevation in PhenoAge (β = 2.96, 95% CI: 2.73–3.18) and a 72% enhanced risk of PhenoAgeAccel (odds ratio [OR] = 1.72, 95% CI: 1.63–1.81). In comparison with the first quartile (Q1), subjects within the fourth quartile (Q4) exhibited an increase of 8.05 years in PhenoAge (β = 8.05, 95% CI: 7.42–8.68) and a 4.33-fold increased probability of PhenoAgeAccel (OR = 4.33, 95% CI: 3.71–5.05). All associations were statistically significant (*P* < .001), with consistent trends (*P*_trend_ < .001). These results establish FLI as a robust predictor of accelerated aging when analyzed as both continuous and categorical variables.

**Figure 4.**
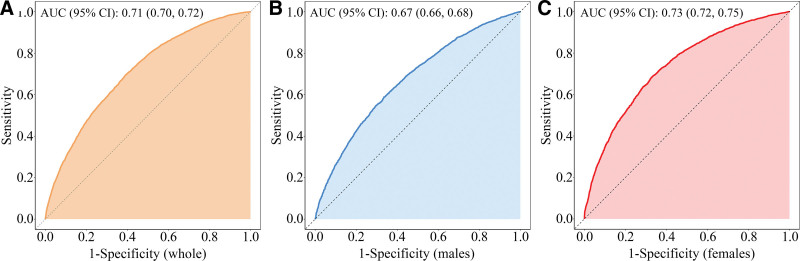
Curves of receiver operating characteristic. Fatty liver index predictive accuracy for the risk of phenotypic age acceleration in the whole population (A), males (B), and females (C). AUC = area under the curve, Cl = confidence interval.

Sex-stratified analyses revealed persistently stronger associations between FLI and BA markers across progressively adjusted models (model 1 to model 3) in females compared to males, with statistically significant interaction effects (*P*_interaction_ < .05). An increase of 1 − SD in FLI was associated with a greater increase in PhenoAge among females (β = 3.33, 95% CI: 3.07–3.58) than among males (β = 2.54, 95% CI: 2.20–2.89) in model 3. Similarly, females exhibited a higher likelihood of PhenoAgeAccel per SD increase in FLI (OR_female_ = 1.89, 95% CI: 1.76–2.03; OR_male_ = 1.57, 95% CI: 1.46–1.69) than males. Quartile-based comparisons further accentuated these sex differences. Females in Q4 demonstrated an increase of 8.99 years in PhenoAge (β = 8.99, 95% CI: 8.25–9.73) and a 5.59-fold increased probability of PhenoAgeAccel (OR = 5.59, 95% CI: 4.57–6.85) relative to Q1 females. In contrast, Q4 males exhibited a 6.78-year increase in PhenoAge (β = 6.78, 95% CI: 5.77–7.79) and a 3.23-fold increased probability of PhenoAgeAccel (OR = 3.23, 95% CI: 2.63–3.97) compared to Q1 males. All reported associations were statistically significant (*P* < .001), with consistent dose–response trends across FLI quartiles (*P*_trend_ < .001). As shown in Figure 4, FLI demonstrated a larger area under the curve (AUC) for PhenoAgeAccel risk prediction in females (AUC = 0.73; 95% CI: 0.72–0.75) than males (AUC = 0.67; 95% CI: 0.66, 0.68) on curves of receiver operating characteristic.

These findings underscored significant sex-specific disparities within the association of FLI with BA. Overall, FLI demonstrated significant positive associations with BA markers. Particularly, in model 3, females exhibited 31% greater increases in PhenoAge (comparing β coefficients: 3.33 vs 2.54) and 20% higher odds of PhenoAgeAccel (comparing ORs: 1.89 vs 1.57) per SD increase in FLI relative to males. The significant interaction effects collectively indicate a substantially stronger association between MASLD and BA in females.

#### 3.2.2. Nonlinear analysis

**Figure 5.**
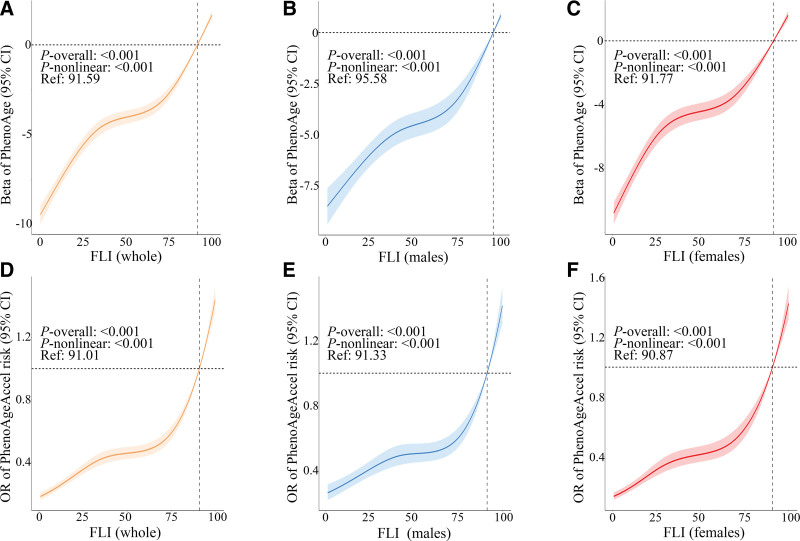
Restricted cubic splines for the association of fatty liver index with biological aging. Associations of fatty liver index (FLI) with both phenotypic age (PhenoAge) and phenotypic age acceleration (PhenoAgeAccel) risk among the whole population (A, D), males (B, E), and females (C, F). CI = confidence interval, FLI = fatty liver index, OR = odds ratio, PhenoAge = phenotypic age, PhenoAgeAccel = phenotypic age acceleration.

Following full adjustment for covariates, RCSs revealed significant nonlinear relationships between FLI and BA across all cohorts (whole population, males, and females; *P*_nonlinear_ < .001). The dose–response curves exhibited a biphasic upward trend, characterized by an initial sustained gradual acceleration of BA followed by a subsequent steep ascent with increasing FLI (Fig. 5). Threshold effect analysis (Table 2) identified FLI thresholds of 91.59 for PhenoAge and 91.01 for PhenoAgeAccel risk in the whole population. Below these thresholds, the FLI-BA association was modest yet significant (β_PhenoAge_ = 0.08, 95% CI: 0.07–0.09; OR_PhenoAgeAccel_ = 1.02, 95% CI: 1.01–1.03). When FLI exceeded the thresholds, the effect sizes increased substantially (β_PhenoAge_ = 0.41, 95% CI: 0.26–0.56; OR_PhenoAgeAccel_ = 1.10, 95% CI: 1.06–1.13; all *P* < .001). Similar biphasic upward patterns were observed in both males and females. Notably, the inflection points (*K*) for the FL-BA relationships were higher in males compared to females (PhenoAge: *K*_male_ = 95.58 vs *K*_female_ = 91.77; PhenoAgeAccel: *K*_male_ = 91.33 vs *K*_female_ = 90.87). However, the magnitudes of the effects before and after the thresholds appeared comparable across the sexes. Likelihood ratio tests demonstrated significantly better models fit for nonlinear compared to linear specifications in all cohorts (*P*_likelihood ratio_ < .001).

**Table 2:** Threshold effect analyses of the association of fatty liver index with biological aging.

Variables	PhenoAge		PhenoAgeAccel	
	β (95% CI)	*P*	OR (95% CI)	*P*
Whole				
Standard linear regression	0.09 (0.08, 0.10)	<.001	1.02 (1.01, 1.03)	<.001
Two-piecewise linear regression	*K* = 91.59		*K* = 91.01	
<*K*	0.08 (0.07, 0.09)	<.001	1.02 (1.01, 1.03)	<.001
≥*K*	0.41 (0.26, 0.56)	<.001	1.10 (1.06, 1.13)	<.001
Likelihood ratio		<.001		<.001
Males				
Standard linear regression	0.07 (0.06, 0.08)	<.001	1.02 (1.01, 1.03)	<.001
Two-piecewise linear regression	*K* = 95.58		*K* = 91.33	
<*K*	0.07 (0.06, 0.08)	<.001	1.02 (1.01, 1.03)	<.001
≥*K*	0.80 (0.30, 1.30)	.002	1.08 (1.03, 1.13)	.002
Likelihood ratio		<.001		<.001
Females				
Standard linear regression	0.11 (0.10, 0.12)	<.001	1.02 (1.01, 1.03)	<.001
Two-piecewise linear regression	*K* = 91.77		*K* = 90.87	
<*K*	0.09 (0.08, 0.10)	<.001	1.02 (1.01, 1.03)	<.001
≥*K*	0.51 (0.29, 0.74)	<.001	1.12 (1.07, 1.18)	<.001
Likelihood ratio		<.001		<.001

Adjusted for demographic covariates, lifestyle behaviors and clinical covariates, *K* = inflection point.

CI = confidence interval, OR = odds ratio, PhenoAge = phenotypic age, PhenoAgeAccel = phenotypic age acceleration.

Nonlinear analyses indicated that FLI exhibited a critical threshold beyond which BA accelerated markedly. Especially, sex-stratified analyses revealed lower thresholds in females than in males. This pattern suggested that females may experience accelerated aging at lower FLI levels than males. Nevertheless, future longitudinal cohort studies remain warranted to establish causal relationships between FLI progression and BA trajectories.

### 3.3. Sensitivity analysis

Robustness was assessed through 3 sensitivity analyses. First, model 3 was further adjusted for depression and sleep disorders. Second, unweighted multivariable regression models were applied. Third, we excluded 1044 participants aged ≥80 years (6.34% of the cohort). The findings of the sensitivity analyses were consistent with those of the primary analysis, suggesting a reliable positive association of FLI with BA among the whole population and sex-stratified cohorts (*P* < .001 for all models), with significant sex interactions (*P*_interaction_ < .01). Sex-stratified analyses confirmed that females exhibited a markedly stronger FLI-BA association than males. All associations demonstrated significant linear trends (*P*_trend_ < .001). For further details, refer to Tables S4–S9, Supplemental Digital Content, https://links.lww.com/MD/Q758. FLI demonstrated higher AUC for PhenoAgeAccel risk prediction in females than males on receiver operating characteristic curves across the 3 sensitivity analyses (Figs. S1–S3, Supplemental Digital Content, https://links.lww.com/MD/Q758).

Reevaluated RCS curves (Figs. S4–S6, Supplemental Digital Content, https://links.lww.com/MD/Q758) and threshold analyses (Tables S10–S12, Supplemental Digital Content, https://links.lww.com/MD/Q758) consistently revealed similar biphasic upward trends (*P*_nonlinear_ < .001) and inflection points across cohorts. The superior fit of piecewise models over linear specifications was maintained in all sensitivity analyses (*P*_likelihood ratio_ < .001). These results further validate the persistence of sex-specific differences in both linear and nonlinear FLI-BA relationships.

### 3.4. Mediation analysis

#### 3.4.1. Association between FLI and METS-IR

Multivariable linear regression analyses employing sequential covariate adjustment revealed a consistently positive association between FLI and METS-IR across all models. As presented in Table 3, the model 3 demonstrated a positive correlation between a 1 − SD elevation in FLI and a 0.81-unit increase in METS-IR (β = 0.81, 95% CI: 0.60–1.02; *P* < .001) in the whole population. Sex-stratified analyses revealed comparable effect sizes (β_male_ = 0.78, 95% CI: 0.56–1.00; β_female_ = 0.72, 95% CI: 0.25–1.20; both *P* < .01), yet a statistically significant sex interaction was observed (*P*_interaction_ < .001; Table S13, Supplemental Digital Content, https://links.lww.com/MD/Q758). This suggested potential sex-based heterogeneity in the FLI-METS-IR relationship despite similar point estimates. A clear dose–response relationship emerged when stratifying FLI into quartiles. Participants in the Q4 exhibited significantly elevated METS-IR levels compared to Q1 (β = 1.93, 95% CI: 1.41–2.46; *P*_trend_ < .001). This pattern persisted in both sexes (β_male_ = 1.87, 95% CI: 1.30–2.43; β_female_ = 1.79, 95% CI: 0.73–2.84; both *P* < .01) with significant linear trends (*P*_trend_ < .01). These results confirmed a robust positive relationship between MASLD severity and IR.

**Table 3:** Associations between fatty liver index and metabolic score for insulin resistance.

Variables	Model 1		Model 2		Model 3	
	β (95% CI)	*P*	β (95% CI)	*P*	β (95% CI)	*P*
Whole population						
Per 1 − SD increase	10.46 (10.32, 10.60)	<.001	10.82 (10.68, 10.97)	<.001	0.81 (0.60, 1.02)	<.001
Quantile						
Q1	Ref		Ref		Ref	
Q2	7.66 (7.41, 7.92)	<.001	8.27 (8.02, 8.52)	<.001	0.35 (0.15, 0.54)	<.001
Q3	14.64 (14.40, 14.88)	<.001	15.45 (15.20, 15.69)	<.001	0.82 (0.52, 1.12)	<.001
Q4	28.39 (27.95, 28.83)	<.001	29.10 (28.64, 29.55)	<.001	1.93 (1.41, 2.46)	<.001
*P*-trend		<.001		<.001		<.001
Males						
Per 1 − SD increase	10.15 (9.95, 10.35)	<.001	10.39 (10.18, 10.60)	<.001	0.78 (0.56, 1.00)	<.001
Quantile						
Q1	Ref		Ref		Ref	
Q2	6.76 (6.38, 7.13)	<.001	7.13 (6.74, 7.51)	<.001	0.44 (0.20, 0.67)	<.001
Q3	13.45 (13.13, 13.77)	<.001	13.95 (13.61, 14.29)	<.001	0.90 (0.57, 1.24)	<.001
Q4	26.68 (26.13, 27.23)	<.001	27.15 (26.57, 27.73)	<.001	1.87 (1.30, 2.43)	<.001
*P*-trend		<.001		<.001		<.001
Females						
Per 1 − SD increase	10.90 (10.69, 11.12)	<.001	11.17 (10.95, 11.39)	<.001	0.72 (0.25, 1.20)	.004
Quantile						
Q1	Ref		Ref		Ref	
Q2	8.21 (7.88, 8.54)	<.001	8.76 (8.42, 9.11)	<.001	0.37 (-.00, 0.75)	.055
Q3	15.63 (15.24, 16.03)	<.001	16.31 (15.90, 16.72)	<.001	0.71 (0.03, 1.39)	.042
Q4	29.99 (29.31, 30.68)	<.001	30.47 (29.76, 31.17)	<.001	1.79 (0.73, 2.84)	.001
*P*-trend		<.001		<.001		.005

Model 1: unadjusted. Model 2: demographic adjusted. Model 3: further adjusted for lifestyle behaviors, clinical covariates, body mass index, and triglycerides.

CI = confidence interval, Q = quartile, Ref = reference, SD = standard deviation.

#### 3.4.2. Association between METS-IR and BA

METS-IR demonstrated robust positive associations with both PhenoAge and PhenoAgeAccel across all regression models. As outlined in Table 4, the application of fully adjusted models indicated that an increase of one SD in METS-IR corresponded to a 4.89-year rise in PhenoAge (β = 4.89, 95% CI: 4.15–5.63) in the whole population. Sex-stratified analyses revealed significantly stronger associations in males (β = 5.23, 95% CI: 4.14–6.32) than in females (β = 4.52, 95% CI: 3.41–5.64), supported by a significant sex interaction (*P*_interaction_ = .009; Table S13, Supplemental Digital Content, https://links.lww.com/MD/Q758). Participants in Q4 of METS-IR showed substantially greater PhenoAge than those in Q1 (β = 6.37, 95% CI: 5.22–7.52; *P*_trend_ < .001). This dose–response pattern was consistent across both sexes (β_male_ = 7.00, 95% CI: 5.48–8.52; β_female_ = 5.50, 95% CI: 3.92–7.08), with significant linear trends (*P*_trend_ < .001). The statistical significance of all associations remained at a level of *P* < .001.

**Table 4:** Associations between metabolic score for insulin resistance and phenotypic age.

Variables	Model 1		Model 2		Model 3	
	β (95% CI)	*P*	β (95% CI)	*P*	β (95% CI)	*P*
Whole population						
Per 1 − SD increase	4.50 (4.17, 4.84)	<.001	3.47 (3.27, 3.67)	<.001	4.89 (4.15, 5.63)	<.001
Quantile						
Q1	Ref		Ref		Ref	
Q2	7.17 (6.24, 8.09)	<.001	2.53 (1.96, 3.10)	<.001	1.85 (1.21, 2.49)	<.001
Q3	9.71 (8.60, 10.82)	<.001	4.48 (3.86, 5.09)	<.001	3.15 (2.43, 3.86)	<.001
Q4	13.57 (12.67, 14.48)	<.001	9.31 (8.68, 9.94)	<.001	6.37 (5.22, 7.52)	<.001
*P*-trend		<.001		<.001		<.001
Males						
Per 1 − SD increase	4.10 (3.51, 4.68)	<.001	3.17 (2.85, 3.50)	<.001	5.23 (4.14, 6.32)	<.001
Quantile						
Q1	Ref		Ref		Ref	
Q2	5.54 (3.99, 7.09)	<.001	1.97 (1.08, 2.87)	<.001	1.72 (0.82, 2.62)	<.001
Q3	8.62 (6.97, 10.27)	<.001	3.74 (2.81, 4.66)	<.001	3.28 (2.32, 4.24)	<.001
Q4	12.73 (11.15, 14.32)	<.001	8.33 (7.36, 9.31)	<.001	7.00 (5.48, 8.52)	<.001
*P*-trend		<.001		<.001		<.001
Females						
Per 1 − SD increase	4.74 (4.34, 5.13)	<.001	3.73 (3.48, 3.97)	<.001	4.52 (3.41, 5.64)	<.001
Quantile						
Q1	Ref		Ref		Ref	
Q2	8.31 (7.09, 9.53)	<.001	2.94 (2.26, 3.62)	<.001	1.87 (1.07, 2.67)	<.001
Q3	10.25 (8.87, 11.63)	<.001	5.08 (4.33, 5.84)	<.001	2.83 (1.77, 3.88)	<.001
Q4	13.84 (12.74, 14.95)	<.001	10.11 (9.37, 10.84)	<.001	5.50 (3.92, 7.08)	<.001
*P*-trend		<.001		<.001		<.001

Model 1: unadjusted. Model 2: demographic adjusted. Model 3: further adjusted for lifestyle behaviors, clinical covariates, body mass index, and triglycerides.

CI = confidence interval, Q = quartile, Ref = reference, SD = standard deviation.

As presented in Table 5, multivariable models consistently demonstrated positive associations of METS-IR with PhenoAgeAccel risk across cohorts. Following full adjustment, each 1 − SD rise in METS-IR corresponded to a 213% higher PhenoAgeAccel risk in the whole population (OR = 3.13, 95% CI: 2.58–3.80). Sex-specific analyses revealed stronger effects in males (OR = 3.23, 95% CI: 2.46–4.24) versus females (OR = 2.98, 95% CI: 2.17–4.08; *P*_interaction_ = .016; Table S13, Supplemental Digital Content, https://links.lww.com/MD/Q758). When analyzed categorically, the highest METS-IR quartile showed substantially elevated risk relative to Q1 (OR = 3.15, 95% CI: 2.38–4.18; *P*_trend_ < .001). This graded relationship persisted in both sexes (OR_male_ = 3.44, 95% CI: 2.48–4.78; OR_female_ = 2.85, 95% CI: 1.86–4.36; *P*_trend_ < .001). All associations remained highly significant (*P* < .001), confirming robust links between IR and advanced BA through both continuous and categorical approaches.

**Table 5:** Associations between metabolic score for insulin resistance and phenotypic age acceleration.

Variables	Model 1		Model 2		Model 3	
	OR (95% CI)	*P*	OR (95% CI)	*P*	OR (95% CI)	*P*
Whole population						
Per 1 − SD increase	2.02 (1.91, 2.12)	<.001	1.98 (1.88, 2.09)	<.001	3.13 (2.58, 3.80)	<.001
Quantile						
Q1	Ref		Ref		Ref	
Q2	1.70 (1.49, 1.95)	<.001	1.54 (1.34, 1.77)	<.001	1.34 (1.15, 1.56)	<.001
Q3	2.51 (2.19, 2.89)	<.001	2.26 (1.96, 2.61)	<.001	1.73 (1.44, 2.08)	<.001
Q4	5.94 (5.11, 6.91)	<.001	5.54 (4.74, 6.48)	<.001	3.15 (2.38, 4.18)	<.001
*P*-trend		<.001		<.001		<.001
Males						
Per 1 − SD increase	1.79 (1.66, 1.92)	<.001	1.84 (1.71, 1.99)	<.001	3.23 (2.46, 4.24)	<.001
Quantile						
Q1	Ref		Ref		Ref	
Q2	1.36 (1.13, 1.64)	.001	1.42 (1.17, 1.72)	<.001	1.34 (1.10, 1.62)	.004
Q3	1.86 (1.54, 2.24)	<.001	1.98 (1.64, 2.40)	<.001	1.79 (1.42, 2.25)	<.001
Q4	4.16 (3.41, 5.07)	<.001	4.50 (3.66, 5.54)	<.001	3.44 (2.48, 4.78)	<.001
*P*-trend		<.001		<.001		<.001
Females						
Per 1 − SD increase	2.16 (2.02, 2.32)	<.001	2.12 (1.97, 2.28)	<.001	2.98 (2.17, 4.08)	<.001
Quantile						
Q1	Ref		Ref		Ref	
Q2	1.69 (1.42, 2.03)	<.001	1.66 (1.38, 1.99)	<.001	1.35 (1.10, 1.67)	.005
Q3	2.66 (2.21, 3.21)	<.001	2.57 (2.11, 3.14)	<.001	1.67 (1.24, 2.25)	.001
Q4	7.10 (5.89, 8.56)	<.001	6.76 (5.57, 8.22)	<.001	2.85 (1.86, 4.36)	<.001
*P*-trend		<.001		<.001		<.001

Model 1: unadjusted. Model 2: demographic adjusted. Model 3: further adjusted for lifestyle behaviors, clinical covariates, body mass index, and triglycerides.

OR = odds ratio, Q = quartile, Ref = reference, SD = standard deviation.

#### 3.4.3. Mediation effect of METS-IR on the association of FLI with BA

**Figure 6.**
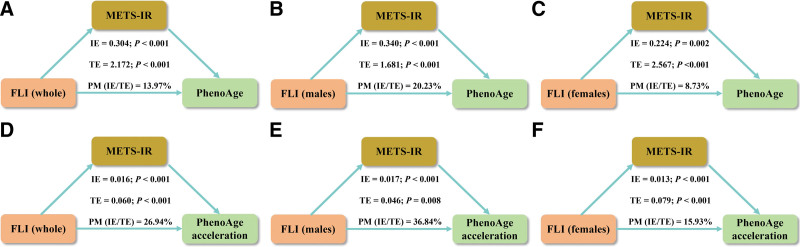
Mediation effects between fatty liver index and biological aging. The mediation effects of METS-IR on the associations of fatty liver index (FLI) with both phenotypic age (PhenoAge) and phenotypic age acceleration (PhenoAgeAccel) risk among the whole population (A, D), males (B, E), and females (C, F). FLI = fatty liver index, IE = indirect effect, METS-IR = metabolic score for insulin resistance, PhenoAge = phenotypic age, PhenoAgeAccel = phenotypic age acceleration, PM = proportion mediated, TE = total effect.

Following standardization of FLI to *Z*-scores (FLI-Z), significant indirect effects of FLI-Z on BA through METS-IR were observed in both the whole population and sex-stratified subgroups after full covariate adjustment (Fig. 6). In the whole cohort, METS-IR accounted for 13.97% (PhenoAge) and 26.94% (PhenoAgeAccel risk) of FLI’s total effect on BA (Table S14, Supplemental Digital Content, https://links.lww.com/MD/Q758). Remarkably, mediation proportions exhibited marked sex-specific variations. Among females, METS-IR explained 8.73% and 15.93% of FLI’s effects on PhenoAge and PhenoAgeAccel risk, respectively. However, males exhibited a substantially higher level of mediation, with 20.23% for PhenoAge and 36.84% for PhenoAgeAccel risk. All indirect pathways achieved statistical significance (*P* < .01), confirming METS-IR as a meaningful mediator in the FLI-BA association, particularly in the male population. Collectively, these findings imply that METS-IR may serve as mediators in biological pathways connecting MASLD to the aging process. However, the validation of these findings necessitates longitudinal studies.

## 4. Discussion

This cross-sectional survey of 16,479 American adults (8264 males and 8215 females) utilized data from the nationally representative NHANES (1999–2020). The results demonstrated robust positive associations between FLI and BA, as measured by PhenoAge and PhenoAgeAccel risk. Significant sex interactions were observed, with females exhibiting 31% stronger FLI-PhenoAge associations and 20% higher PhenoAgeAccel risk compared to males. Nonlinear analyses, through RCS curves and threshold analyses, revealed a biphasic upward trend in which BA showed sustained gradual acceleration followed by a steep ascent beyond sex-specific FLI thresholds, with consistently lower inflection points in females than in males. These findings remained consistent in several sensitivity analyses. Mediation analyses identified METS-IR as a potential mediator, accounting for 13.97% to 26.94% of FLI-BA associations overall, with significant sex interactions in both FLI-METS-IR and METS-IR-BA pathways. METS-IR contributed substantially more to BA in males (PhenoAge: 20.2%; PhenoAgeAccel: 36.8%) than in females (8.7%; 15.9%). These results elucidate sex-dimorphic mechanisms linking MASLD to accelerated aging through IR, informing the design of sex-specific public health strategies aimed at MASLD and aging. However, longitudinal validation remains warranted.

Our study demonstrated that FLI and BA share a robust positive association, which intensifies significantly once FLI values exceed approximately 90 units. These findings align consistently with prior epidemiological and pathophysiological evidence establishing a close link between MASLD and accelerated aging processes. Epidemiologically, the prevalence of MASLD surges to approximately 40% in those aged 60 years or older, markedly exceeding the rate of 25% observed in the general adult population. Furthermore, elderly patients exhibit a heightened susceptibility to progression toward metabolic dysfunction-associated steatohepatitis and hepatic fibrosis.^[32]^ Pathophysiologically, MASLD is established as a systemic disorder, exerting profound effects across multiple organ systems. It significantly impacts aging-related conditions, including DM, CVD, and CKD.^[33–35]^ Collectively, this body of evidence underscores the predictive significance of FLI, a well-established indicator of MASLD burden, as a marker for accelerated BA. However, conducting longitudinal studies is necessary to verify causal relationships.

IR represents a pathological condition characterized by a diminished biological response to insulin. METS-IR serves as a robust indicator for assessing IR.^[36]^ Our findings regarding the association between FLI and BA are consistent with prior research;^[37]^ however, mediation analysis in our study further revealed that METS-IR significantly mediated 13.97% to 26.94% of this association. Evidence indicates that IR, initiated by MASLD, ultimately drives BA through multiple underlying mechanisms including dysregulated energy metabolism, chronic inflammation, oxidative stress, vascular dysfunction, and telomere shortening. Specifically, excessive adipose tissue expansion originating from the liver triggers systemic low-grade inflammation via macrophage infiltration and proinflammatory cytokine secretion. The cytokines have been demonstrated to activate JNK/IKKβ kinases kinases. Furthermore, these cytokines suppress insulin receptor substrate phosphorylation and impair the PI3K/Akt signaling pathway. The result of this disruption is impaired glucose uptake and, by consequence, the promotion of IR.^[38]^ Concurrently, lipotoxicity, characterized by ceramide and diacylglycerol accumulation, induces mitochondrial fragmentation and oxidative stress, exacerbating aberrant fat deposition in hepatic tissues and further disrupting insulin-mediated glucose metabolism.^[39,40]^ Subsequently, IR accelerates aging through multiple pathways. Firstly, IR induces metabolic dysregulation via mitochondrial dysfunction and endoplasmic reticulum stress. This involves hepatic mitochondrial remodeling, increased pyruvate dehydrogenase activity, and upregulated pyruvate carboxylase, disrupting tricarboxylic acid cycle homeostasis and elevating reactive oxygen species accumulation, triggering deoxyribonucleic acid damage and senescence.^[41]^ Concurrently, IR-triggered sustained unfolded protein response activation disrupts endoplasmic reticulum-mitochondria contacts, promoting calcium overload and apoptosis.^[20]^ Secondly, IR impairs monocyte phagocytic and lysosomal function, weakening immune surveillance. This fosters chronic inflammation and senescence-associated secretory phenotype factors accumulation, while enhanced phagocytic function in long-lived individuals correlates with insulin sensitivity.^[42]^ Thirdly, IR impedes PI3K/Akt signaling, reducing antioxidant defenses and increasing reactive oxygen species-mediated macromolecular damage (deoxyribonucleic acid, proteins, lipids).^[43]^ Fourthly, hyperinsulinemia activates renin-angiotensin-aldosterone system, promoting vascular calcification, endothelial dysfunction, reduced cardiac output, and systemic ischemia-hypoxia; reduced insulin sensitivity significantly increases accelerated aging risk, partly mediated by arterial stiffness.^[44]^ Fifthly, fluctuating glucose/insulin during IR activates JAK/STAT signaling, upregulating telomerase inhibitors and suppressing telomerase activity.^[45]^ Collectively, these multifaceted pathways, spanning metabolic dysregulation, inflammation, oxidative damage, vascular compromise, and telomeric attrition, establish IR as a vital mediator linking MASLD to accelerated BA. However, longitudinal studies are required to elucidate the causality and deeper mechanisms underlying these relationships.

Beyond IR, MASLD may accelerate aging through chronic inflammation. This inflammation induces cellular damage via apoptosis and impaired tissue repair, while promoting oxidative stress and metabolic dysregulation.^[46]^ Proinflammatory cytokines from MASLD disrupt gut microbiota homeostasis and compromise intestinal barrier integrity, exacerbating systemic inflammation and immune dysfunction in a vicious cycle.^[47]^ Modifiable lifestyle factors (e.g., inactivity, smoking, alcohol) associated with MASLD independently engage aging pathways.^[48]^ Additionally, hepatic lipid accumulation triggers epigenetic reprogramming, such as steatosis-induced upregulation of *BAZ2B*, which suppresses peroxisome proliferator-activated receptor alpha signaling and fatty acid oxidation, directly promoting hepatocyte senescence.^[49]^ Future research should further elucidate MASLD-aging mechanisms.

Our data indicate that the association between FLI and late-stage BA is significantly stronger in females than in males, which was consistent with previous studies showing sex-specific associations between FLI and age-related diseases.^[17,21]^ This suggests MASLD may promote aging through sex-dimorphic mechanisms. While METS-IR demonstrated comparable mediating effects across different sexes, its contribution to the FLI-BA association in females was less than half that observed in males. This finding suggests that females may be more dependent on non-IR mediating factors. The potential mechanisms include: Estrogen-driven metabolic reprogramming. Fluctuations during reproductive transitions alter hepatic lipid metabolism, increasing hydrophobic bile acids that trigger inflammation and oxidative stress.^[50]^ Enhanced cardiovascular inflammation sensitivity. Adipose-driven inflammatory responses have been demonstrated to induce more severe endothelial dysfunction in females. Moreover, excessive adipose accumulation has been shown to be strongly associated with the risk of carotid atherosclerosis, but this association does not exist in males.^[51,52]^ Epigenetic dysregulation. Estrogen variations may potentiate *BAZ2B* accumulation, suppressing peroxisome proliferator-activated receptor alpha activity to accelerate hepatocyte senescence.^[14,49]^ Gut microbiota dysbiosis. Research has identified that females diagnosed with fatty liver disease exhibit an increase in *Fusobacterium/Escherichia-Shigella* bacteria and a decrease in butyrate producers within their gut microbiota. This results in a decrease in the production of 7-ketocholic acid, which in turn causes damage to the intestinal barrier function and promotes endotoxin-driven systemic inflammation.^[50]^ Although the precise mechanisms require further elucidation, this evidence highlights the necessity for prospective studies to determine the MASLD-aging causal link.

This investigation demonstrates several key strengths. First, it is the first to identify the previously unrecognized association between FLI and BA (assessed via PhenoAge and PhenoAgeAccel), while establishing the mediating role of METS-IR in these relationships and revealing pronounced sex-specific disparities. Second, our utilization of NHANES’ complex probability-based sampling design ensures statistically robust nationwide generalizability. Third, these associations remained consistent across multivariable-adjusted models and sensitivity analyses, confirming their robustness. Our findings position FLI as a promising sex-stratified biomarker of BA, generating testable hypotheses for future longitudinal studies to validate its predictive capacity for aging trajectories and explore sex-targeted preventive strategies. Notwithstanding these contributions, 4 limitations should be acknowledged. First, this cross-sectional approach compromises causal inference on the temporal sequence between FLI and BA, particularly for sex-specific mechanisms; prospective cohort studies or experimental interventions are required to establish causality. Second, despite stringent control for multiple confounders, residual unmeasured confounding (such as genetic/epigenetic predispositions, occupational exposures) cannot be excluded, underscoring the need for future multi-omics integration to enhance confounding control. Third, the exclusive reliance on American-based NHANES data restricts generalizability to other populations, necessitating external validation in ethnically diverse cohorts. Finally, while PhenoAge captures multidimensional aging features, incorporating complementary biomarkers (such as homeostatic dysregulation indices, allostatic load quantification, and Klemera–Doubal method-derived metrics) in subsequent research could deepen mechanistic understanding of MASLD-aging interrelationships.

## 5. Conclusions

This study demonstrates a significant positive association between FLI and BA. The association was stronger in females, with a greater proportion of the effect mediated by METS-IR in males. These outcomes emphasize the requirement for public policy strategies that target MASLD and aging in a sex-specific manner. Prospective studies are required to establish causality and elucidate the sex-dimorphic biological mechanisms underlying these associations.

## Acknowledgments

We thank the NHANES program for providing public data.

## Author contributions

**Data curation**: Haifeng Liu, Tiejun Liu.

**Formal analysis**: Jia Yang, Haifeng Liu.

**Methodology**: Jia Yang, Haifeng Liu.

**Project administration**: Jia Yang.

**Validation**: Jia Yang, Weimin Zhao, Tiejun Liu.

**Visualization**: Jia Yang, Haifeng Liu, Weimin Zhao.

**Writing – original draft**: Jia Yang, Haifeng Liu.

**Writing – review & editing**: Jia Yang, Weimin Zhao, Tiejun Liu.

## Supplementary Material
