# Supplementary material for: Sex differences in the association between fatty liver index and biological aging: A mediation analysis of insulin resistance in a cross-sectional study
Source: Medicine (Baltimore). 2026 May 12;104(49):e46152. doi: 10.1097/MD.0000000000046152 (PMC12689076; doi:10.1097/MD.0000000000046152)
Supplement: Supplementary file 1 [file medi-104-e46152-s001.docx]

**Table S1.** Formulae for phenotypic age.

| Formulae | $\text{Phenotypic age }=141.50+\frac{Ln\left[-0.00553\times L\text{n}\left(\text{exp}\left(\frac{\left(\text{-1.51714}\times\text{exp}\left(\text{xb}\right)\right)}{0.0076927}\right)\right)\right]}{0.09165}$ |
| --- | --- |
| xb | −19.907−0.0336×Albumin+0.0095×Creatinine+0.1953×Glucose+0.0954×LnCRP−0.0120×Lymphocyte Percentage+0.0268×Mean Cell Volume + 0.00188×Alkaline Phosphatase+0.0554×Leukocyte Count+0.0804×chronological age. |

**Table S2.** Definitions of clinical covariates.

| **Covariates** | **Abbreviation** | **Diagnostic Criteria** | **Data Source** | **References** |
| --- | --- | --- | --- | --- |
| Hypertension | HTN | The diagnosis is based on one of = | Physical examination + Medical history questionnaire | PMID = 39834472 |
|  |  | 1) Systolic blood pressure >140 mmHg or diastolic blood pressure >90 mmHg |  |  |
|  |  | 2) Physician-confirmed diagnosis |  |  |
| Cardiovascular Disease | CVD | The diagnosis is based on one of = | Medical history questionnaire | PMID = 40022176 |
|  |  | 1) Congestive heart failure |  |  |
|  |  | 2) Coronary artery disease |  |  |
|  |  | 3) Myocardial infarction |  |  |
|  |  | 4) Angina pectoris |  |  |
|  |  | 5) Stroke |  |  |
| Diabetes Mellitus | DM | The diagnosis is based on one of = | Laboratory tests + Medical history questionnaire | PMID = 39696515 |
|  |  | 1) Glycated hemoglobin ≥6.5% |  |  |
|  |  | 2) Fasting glucose ≥126 mg/dL |  |  |
|  |  | 3) 2-h oral glucose tolerance test ≥200 mg/dL |  |  |
|  |  | 4) Current use of antidiabetic agents |  |  |
|  |  | 5) A previous diagnosis by a physician |  |  |
| Chronic Kidney Disease | CKD | The diagnosis is based on one of = | Laboratory tests | PMID = 40177175 |
|  |  | 1) Estimated glomerular filtration rate <60 mL/min/1.73 m² (CKD-EPI equation) |  |  |
|  |  | 2) Urine albumin-creatinine ratio >30 mg/g |  |  |
| Cancer | - | Cancer is defined as having been told by a doctor or other healthcare professional that a malignant neoplasm has been diagnosed. | Medical history questionnaire | PMID = 40022176 |

**Table S3.** Sex-specific associations of fatty liver index quartiles with biological aging.

| Males | Total (n = 8,264) | Q1 (n=2,026) | Q2 (n=2,152) | Q3 (n=2,065) | Q4 (n=2,021) | Statistic | *P* |
| --- | --- | --- | --- | --- | --- | --- | --- |
| Phenotypic age (years) | 56.93 (44.05, 71.07) | 46.82 (34.76, 60.96) | 56.65 (43.62, 72.07) | 59.88 (48.60, 71.79) | 63.83 (51.67, 75.80) | χ²=357.36 | < .001 |
| Phenotypic age acceleration, n (%) |  | | | | | χ²=407.49 | < .001 |
| No | 3,955 (50.96) | 1,234 (64.25) | 1,128 (55.43) | 934 (50.44) | 659 (33.75) |  |  |
| Yes | 4,309 (49.04) | 792 (35.75) | 1,024 (44.57) | 1,131 (49.56) | 1,362 (66.25) |  |  |
| Females | Total (n = 8,215) | Q1 (n=1,691) | Q2 (n=2,006) | Q3 (n=2,291) | Q4 (n=2,227) | Statistic | *P* |
| Phenotypic age (years) | 55.67 (42.71, 68.69) | 43.33 (33.56, 53.49) | 54.33 (42.52, 66.48) | 60.65 (48.84, 72.69) | 64.37 (52.58, 74.39) | χ²=1015.49 | < .001 |
| Phenotypic age acceleration, n (%) |  | | | | | χ²=833.81 | < .001 |
| No | 5,068 (65.37) | 1,348 (82.16) | 1,457 (74.71) | 1,394 (62.54) | 869 (42.09) |  |  |
| Yes | 3,147 (34.63) | 343 (17.84) | 549 (25.29) | 897 (37.46) | 1,358 (57.91) |  |  |

The continuous variables demonstrated non-normal distributions as assessed by the Kolmogorov-Smirnov test. Consequently, comparisons between the groups utilized a weighted Kruskal-Wallis test, with results expressed as median values (first quartile, third quartile). Categorical variables were compared through weighted chi-square tests and expressed as counts (unweighted) as well as proportions (weighted).

**Supplementary Table S4.** Associations of fatty liver index with biological aging after additional adjustment for depression and sleep disorders.

| **Variables** | **PhenoAge** | | **PhenoAgeAccel** | |
| --- | --- | --- | --- | --- |
|  | **β (95% CI)** | ***P*** | **OR (95% CI)** | ***P*** |
| Whole population | | | | |
| Per 1–SD increase | 2.94 (2.72, 3.17) | < .001 | 1.72 (1.63, 1.81) | < .001 |
| Quantile |  | | | |
| Q1 | 0.00 (Reference) |  | 1.00 (Reference) |  |
| Q2 | 2.90 (2.29, 3.52) | < .001 | 1.65 (1.44, 1.90) | < .001 |
| Q3 | 4.86 (4.25, 5.46) | < .001 | 2.33 (1.99, 2.73) | < .001 |
| Q4 | 8.01 (7.38, 8.63) | < .001 | 4.31 (3.70, 5.03) | < .001 |
| P-trend |  | < .001 |  | < .001 |
| Males | | | | |
| Per 1–SD increase | 2.54 (2.19, 2.88) | < .001 | 1.55 (1.45, 1.66) | < .001 |
| Quantile |  | | | |
| Q1 | 0.00 (Reference) |  | 1.00 (Reference) |  |
| Q2 | 1.86 (0.95, 2.77) | < .001 | 1.39 (1.16, 1.67) | < .001 |
| Q3 | 3.81 (2.84, 4.78) | < .001 | 1.79 (1.44, 2.23) | < .001 |
| Q4 | 6.78 (5.77, 7.78) | < .001 | 3.24 (2.64, 3.97) | < .001 |
| P-trend |  | < .001 |  | < .001 |
| Females | | | | |
| Per 1–SD increase | 3.30 (3.04, 3.56) | < .001 | 1.89 (1.76, 2.03) | < .001 |
| Quantile |  | | | |
| Q1 | 0.00 (Reference) |  | 1.00 (Reference) |  |
| Q2 | 3.66 (2.86, 4.46) | < .001 | 1.91 (1.59, 2.31) | < .001 |
| Q3 | 5.61 (4.95, 6.26) | < .001 | 2.97 (2.45, 3.60) | < .001 |
| Q4 | 8.93 (8.18, 9.68) | < .001 | 5.56 (4.54, 6.81) | < .001 |
| P-trend |  | < .001 |  | < .001 |

The models were adjusted for age, sex (only in the model of the whole population), race, education, marital status, poverty status, smoking status, alcohol consumption, physical activity, HTN, DM, CVD, cancer, CKD, depression, and sleep disorders. PhenoAge = phenotypic age, PhenoAgeAccel = phenotypic age acceleration, Cl = confidence interval, OR = odds ratio, SD = standard deviation.

**Table S5.** Sex interaction analysis after additional adjustment for depression and sleep disorders.

|  | **Associations between FLI and PhenoAge** | | | **Associations between FLI and PhenoAgeAccel risk** | | |
| --- | --- | --- | --- | --- | --- | --- |
|  | **β (95% CI)** | ***P*-value** | ***P* for interaction** | **OR (95% CI)** | ***P*-value** | ***P* for interaction** |
| Males | 2.54 (2.19, 2.88) | < .001 | .007 | 1.55 (1.45, 1.66) | < .001 | .001 |
| Females | 3.30 (3.04, 3.56) | < .001 |  | 1.89 (1.76, 2.03) | < .001 |  |

The models were adjusted for age, race, education, marital status, poverty status, smoking status, alcohol consumption, physical activity, HTN, DM, CVD, cancer, CKD, depression, and sleep disorders. FLI = fatty liver index, BA = biological aging, PhenoAge = phenotypic age, PhenoAgeAccel = phenotypic age acceleration, CI = confidence interval, OR = odds ratio.

**Table S6.** Associations between fatty liver index and biological aging with unweighted multivariable regression.

| **Variables** | **PhenoAge** | | **PhenoAgeAccel** | |
| --- | --- | --- | --- | --- |
|  | **β (95% CI)** | ***P*** | **OR (95% CI)** | ***P*** |
| Whole population | | | | |
| Per 1–SD increase | 2.88 (2.73, 3.04) | < .001 | 1.71 (1.65, 1.77) | < .001 |
| Quantile |  | | | |
| Q1 | 0.00 (Reference) |  | 1.00 (Reference) |  |
| Q2 | 2.54 (2.13, 2.96) | < .001 | 1.57 (1.42, 1.73) | < .001 |
| Q3 | 4.82 (4.39, 5.24) | < .001 | 2.35 (2.12, 2.60) | < .001 |
| Q4 | 7.60 (7.16, 8.04) | < .001 | 4.11 (3.70, 4.57) | < .001 |
| P-trend |  | < .001 |  | < .001 |
| Males | | | | |
| Per 1–SD increase | 2.35 (2.12, 2.58) | < .001 | 1.55 (1.47, 1.63) | < .001 |
| Quantile |  | | | |
| Q1 | 0.00 (Reference) |  | 1.00 (Reference) |  |
| Q2 | 1.70 (1.05, 2.35) | < .001 | 1.40 (1.21, 1.62) | < .001 |
| Q3 | 3.91 (3.26, 4.55) | < .001 | 1.99 (1.73, 2.31) | < .001 |
| Q4 | 6.01 (5.33, 6.68) | < .001 | 3.11 (2.67, 3.63) | < .001 |
| P-trend |  | < .001 |  | < .001 |
| Females | | | | |
| Per 1–SD increase | 3.40 (3.20, 3.61) | < .001 | 1.90 (1.80, 2.00) | < .001 |
| Quantile |  | | | |
| Q1 | 0.00 (Reference) |  | 1.00 (Reference) |  |
| Q2 | 3.19 (2.65, 3.74) | < .001 | 1.74 (1.50, 2.01) | < .001 |
| Q3 | 5.52 (4.95, 6.09) | < .001 | 2.76 (2.38, 3.19) | < .001 |
| Q4 | 9.08 (8.49, 9.67) | < .001 | 5.48 (4.72, 6.37) | < .001 |
| P-trend |  | < .001 |  | < .001 |

The models were adjusted for age, sex (only in the model of the whole population), race, education, marital status, poverty status, smoking status, alcohol consumption, physical activity, HTN, DM, CVD, cancer, and CKD. FLI = fatty liver index, BA = biological aging, PhenoAge = phenotypic age, PhenoAgeAccel = phenotypic age acceleration, Cl = confidence interval, OR = odds ratio, SD = standard deviation, Q = quantile.

**Table S7.** Sex interaction analysis with unweighted multivariable regression.

|  | **Associations between FLI and PhenoAge** | | | **Associations between FLI and PhenoAgeAccel risk** | | |
| --- | --- | --- | --- | --- | --- | --- |
|  | **β (95% CI)** | ***P*-value** | ***P* for interaction** | **OR (95% CI)** | ***P*-value** | ***P* for interaction** |
| Males | 2.35 (2.12, 2.58) | < .001 | < .001 | 1.55 (1.47, 1.63) | < .001 | < .001 |
| Females | 3.40 (3.20, 3.61) | < .001 |  | 1.90 (1.80, 2.00) | < .001 |  |

The models were adjusted for age, race, education, marital status, poverty status, smoking status, alcohol consumption, physical activity, HTN, DM, CVD, cancer, and CKD. FLI = fatty liver index, BA = biological aging, PhenoAge = phenotypic age, PhenoAgeAccel = phenotypic age acceleration, CI = confidence interval, OR = odds ratio.

**Table S8.** Associations between fatty liver index and biological aging following exclusion of participants aged ≥80 years.

| **Variables** | **PhenoAge** | | **PhenoAgeAccel** | |
| --- | --- | --- | --- | --- |
|  | **β (95% CI)** | ***P*** | **OR (95% CI)** | ***P*** |
| Whole population | | | | |
| Per 1–SD increase | 3.02 (2.78, 3.26) | < .001 | 1.73 (1.64, 1.83) | < .001 |
| Quantile |  | | | |
| Q1 | 0.00 (Reference) |  | 1.00 (Reference) |  |
| Q2 | 2.98 (2.34, 3.61) | < .001 | 1.68 (1.46, 1.94) | < .001 |
| Q3 | 5.01 (4.35, 5.66) | < .001 | 2.38 (2.01, 2.82) | < .001 |
| Q4 | 8.20 (7.53, 8.86) | < .001 | 4.45 (3.79, 5.22) | < .001 |
| P-trend |  | < .001 |  | < .001 |
| Males | | | | |
| Per 1–SD increase | 2.58 (2.22, 2.94) | < .001 | 1.56 (1.45, 1.68) | < .001 |
| Quantile |  | | | |
| Q1 | 0.00 (Reference) |  | 1.00 (Reference) |  |
| Q2 | 1.84 (0.88, 2.80) | < .001 | 1.39 (1.14, 1.68) | < .001 |
| Q3 | 3.85 (2.83, 4.87) | < .001 | 1.80 (1.43, 2.27) | < .001 |
| Q4 | 6.87 (5.82, 7.91) | < .001 | 3.26 (2.64, 4.02) | < .001 |
| P-trend |  | < .001 |  | < .001 |
| Females | | | | |
| Per 1–SD increase | 3.42 (3.14, 3.69) | < .001 | 1.93 (1.79, 2.08) | < .001 |
| Quantile |  | | | |
| Q1 | 0.00 (Reference) |  | 1.00 (Reference) |  |
| Q2 | 3.81 (3.00, 4.63) | < .001 | 1.99 (1.64, 2.41) | < .001 |
| Q3 | 5.85 (5.14, 6.56) | < .001 | 3.11 (2.53, 3.83) | < .001 |
| Q4 | 9.22 (8.43, 10.00) | < .001 | 5.93 (4.80, 7.32) | < .001 |
| P-trend |  | < .001 |  | < .001 |

The models were adjusted for age, sex (only in the model of the whole population), race, education, marital status, poverty status, smoking status, alcohol consumption, physical activity, HTN, DM, CVD, cancer, and CKD. PhenoAge = phenotypic age, PhenoAgeAccel = phenotypic age acceleration, Cl = confidence interval, OR = odds ratio, SD = standard deviation, Q = quantile.

**Table S9.** Sex interaction analysis following exclusion of participants aged ≥80 years.

|  | **Associations between FLI and PhenoAge** | | | **Associations between FLI and PhenoAgeAccel risk** | | |
| --- | --- | --- | --- | --- | --- | --- |
|  | **β (95% CI)** | ***P*-value** | ***P* for interaction** | **OR (95% CI)** | ***P*-value** | ***P* for interaction** |
| Males | 2.58 (2.22, 2.94) | < .001 | .007 | 1.56 (1.45, 1.67) | < .001 | .002 |
| Females | 3.42 (3.14, 3.69) | < .001 |  | 1.93 (1.79, 2.08) | < .001 |  |

The models were adjusted for age, race, education, marital status, poverty status, smoking status, alcohol consumption, physical activity, HTN, DM, CVD, cancer, and CKD. FLI = fatty liver index, BA = biological aging, PhenoAge = phenotypic age, PhenoAgeAccel = phenotypic age acceleration, CI = confidence interval, OR = odds ratio.

**Table S10.** Threshold effect analysis between fatty liver index and biological aging after additional adjustment for depression and sleep disorders.

| **Variables** | **PhenoAge** | | **PhenoAgeAccel** | |
| --- | --- | --- | --- | --- |
|  | **β (95% CI)** | ***P*** | **OR (95% CI)** | ***P*** |
| Whole population |  | | | |
| Standard linear regression | 0.09 (0.08, 0.09) | < .001 | 1.02 (1.02, 1.02) | < .001 |
| Two-piecewise linear regression | K = 91.59 | | K = 91.01 | |
| <K | 0.08 (0.07, 0.08) | < .001 | 1.01 (1.01, 1.02) | < .001 |
| ≥K | 0.41 (0.26, 0.56) | < .001 | 1.10 (1.06, 1.13) | < .001 |
| Likelihood ratio |  | < .001 |  | < .001 |
| Males |  | | | |
| Standard linear regression | 0.07 (0.07, 0.08) | < .001 | 1.01 (1.01, 1.02) | < .001 |
| Two-piecewise linear regression | K = 95.58 | | K = 91.33 | |
| <K | 0.07 (0.06, 0.07) | < .001 | 1.01 (1.01, 1.01) | < .001 |
| ≥K | 0.78 (0.28, 1.28) | 0.002 | 1.08 (1.03, 1.13) | 0.002 |
| Likelihood ratio |  | < .001 |  | < .001 |
| Females |  | | | |
| Standard linear regression | 0.11 (0.10, 0.11) | < .001 | 1.02 (1.02, 1.02) | < .001 |
| Two-piecewise linear regression | K = 91.77 | | K = 90.88 | |
| <K | 0.09 (0.08, 0.10) | < .001 | 1.02 (1.01, 1.02) | < .001 |
| ≥K | 0.52 (0.29, 0.75) | < .001 | 1.12 (1.07, 1.18) | < .001 |
| Likelihood ratio |  | < .001 |  | < .001 |

The models were adjusted for age, sex (only in the model of the whole population), race, education, marital status, poverty status, smoking status, alcohol consumption, physical activity, HTN, DM, CVD, cancer, CKD, depression, and sleep disorders. PhenoAge = phenotypic age, PhenoAgeAccel = phenotypic age acceleration., CI = confidence interval, OR = odds ratio, K = inflection point.

**Table S11.** Threshold effect analysis between fatty liver index and biological aging with unweighted multivariable regression.

| **Variables** | **PhenoAge** | | **PhenoAgeAccel** | |
| --- | --- | --- | --- | --- |
|  | **β (95% CI)** | ***P*** | **OR (95% CI)** | ***P*** |
| Whole population |  | | | |
| Standard linear regression | 0.09 (0.09, 0.10) | < .001 | 1.02 (1.02, 1.02) | < .001 |
| Two-piecewise linear regression | K = 91.59 | | K = 91.01 | |
| <K | 0.08 (0.07, 0.08) | < .001 | 1.01 (1.01, 1.02) | < .001 |
| ≥K | 0.41 (0.26, 0.56) | < .001 | 1.10 (1.06, 1.13) | < .001 |
| Likelihood ratio |  | < .001 |  | < .001 |
| Males |  | | | |
| Standard linear regression | 0.07 (0.07, 0.08) | < .001 | 1.01 (1.01, 1.02) | < .001 |
| Two-piecewise linear regression | K = 95.58 | | K = 91.33 | |
| <K | 0.07 (0.06, 0.07) | < .001 | 1.01 (1.01, 1.01) | < .001 |
| ≥K | 0.80 (0.30, 1.30) | 0.002 | 1.08 (1.03, 1.13) | 0.002 |
| Likelihood ratio |  | < .001 |  | < .001 |
| Females |  | | | |
| Standard linear regression | 0.11 (0.10, 0.11) | < .001 | 1.02 (1.02, 1.02) | < .001 |
| Two-piecewise linear regression | K = 91.77 | | K = 90.87 | |
| <K | 0.09 (0.08, 0.10) | < .001 | 1.02 (1.01, 1.02) | < .001 |
| ≥K | 0.51 (0.29, 0.74) | < .001 | 1.12 (1.07, 1.18) | < .001 |
| Likelihood ratio |  | < .001 |  | < .001 |

The models were adjusted for age, sex (only in the model of the whole population), race, education, marital status, poverty status, smoking status, alcohol consumption, physical activity, HTN, DM, CVD, cancer, and CKD. PhenoAge = phenotypic age, PhenoAgeAccel = phenotypic age acceleration, CI = confidence interval, OR = odds ratio, K = inflection point.

**Table S12.** Threshold effect analysis between fatty liver index and biological aging following exclusion of participants aged ≥80 years.

| **Variables** | **PhenoAge** | | **PhenoAgeAccel** | |
| --- | --- | --- | --- | --- |
|  | **β (95% CI)** | ***P*** | **OR (95% CI)** | ***P*** |
| Whole population |  | | | |
| Standard linear regression | 0.09 (0.09, 0.10) | < .001 | 1.02 (1.02, 1.02) | < .001 |
| Two-piecewise linear regression | K = 91.57 | | K = 90.93 | |
| <K | 0.08 (0.07, 0.09) | < .001 | 1.01 (1.01, 1.02) | < .001 |
| ≥K | 0.42 (0.27, 0.58) | < .001 | 1.10 (1.06, 1.14) | < .001 |
| Likelihood ratio |  | < .001 |  | < .001 |
| Males |  | | | |
| Standard linear regression | 0.07 (0.07, 0.08) | < .001 | 1.01 (1.01, 1.02) | < .001 |
| Two-piecewise linear regression | K = 95.74 | | K = 91.01 | |
| <K | 0.07 (0.06, 0.08) | < .001 | 1.01 (1.01, 1.01) | < .001 |
| ≥K | 0.88 (0.34, 1.42) | 0.001 | 1.08 (1.03, 1.14) | < .001 |
| Likelihood ratio |  | < .001 |  | < .001 |
| Females |  | | | |
| Standard linear regression | 0.11 (0.10, 0.12) | < .001 | 1.02 (1.02, 1.02) | < .001 |
| Two-piecewise linear regression | K = 91.78 | | K = 90.82 | |
| <K | 0.10 (0.09, 0.11) | < .001 | 1.02 (1.02, 1.02) | < .001 |
| ≥K | 0.53 (0.30, 0.76) | < .001 | 1.13 (1.07, 1.18) | < .001 |
| Likelihood ratio |  | < .001 |  | < .001 |

The models were adjusted for age, sex (only in the model of the whole population), race, education, marital status, poverty status, smoking status, alcohol consumption, physical activity, HTN, DM, CVD, cancer, and CKD. PhenoAge = phenotypic age, PhenoAgeAccel = phenotypic age acceleration, CI = confidence interval, OR = odds ratio, K = inflection point.

**Table S13.** Sex interaction analyses.

| **Sex** | **Associations between FLI and METS-IR** | | | **Associations between METS-IR and PhenoAge** | | | **Associations between METS-IR and PhenoAgeAccel risk** | | |
| --- | --- | --- | --- | --- | --- | --- | --- | --- | --- |
|  | **β (95% CI)** | ***P*** | ***P* for interaction** | **β (95% CI)** | ***P*** | ***P* for interaction** | **OR (95% CI)** | ***P*** | ***P* for interaction** |
| Males | 0.78 (0.56, 1.00) | < .001 | < .001 | 5.21 (4.12, 6.30) | < .001 | 0.009 | 3.21 (2.44, 4.22) | < .001 | .016 |
| Females | 0.72 (0.24, 1.20) | .004 |  | 4.52 (3.41, 5.63) | < .001 |  | 2.98 (2.17, 4.08) | < .001 |  |

The models were adjusted for age, race, education, marital status, poverty status, smoking status, alcohol consumption, physical activity, HTN, DM, CVD, cancer, CKD, BMI, and TG. FLI = fatty liver index, METS-IR = metabolic score for insulin resistance, PhenoAge = phenotypic age, PhenoAgeAccel = phenotypic age acceleration, CI = confidence interval, OR = odds ratio.

**Table S14.** Mediating effects of metabolic score for insulin resistance.

| **Effect** | **PhenoAge** | | | **PhenoAgeAccel** | | |
| --- | --- | --- | --- | --- | --- | --- |
|  | **β (95% CI)** | ***P*** | **Proportion of mediation** | **β (95% CI)** | ***P*** | **Proportion of mediation** |
| Whole population |  | | | | | |
| Indirect | 0.304 (0.215, 0.378) | < .001 | 13.97% | 0.016 (0.011, 0.021) | < .001 | 26.94% |
| Direct | 1.869 (1.336, 2.056) | < .001 |  | 0.044 (0.020, 0.070) | < .001 |  |
| Total | 2.172 (1.643, 2.357) | < .001 |  | 0.060 (0.035, 0.086) | < .001 |  |
| Males |  |  |  |  |  |  |
| Indirect | 0.340 (0.214, 0.422) | < .001 | 20.23% | 0.017 (0.011, 0.023) | < .001 | 36.84% |
| Direct | 1.341 (0.928, 1.993) | < .001 |  | 0.029 (0.004, 0.055) | .002 |  |
| Total | 1.681 (1.250, 2.298) | < .001 |  | 0.046 (0.012, 0.082) | .008 |  |
| Females |  |  |  |  |  |  |
| Indirect | 0.224 (0.112, 0.375) | .002 | 8.73% | 0.013 (0.005, 0.023) | < .001 | 15.93% |
| Direct | 2.343 (1.530, 2.437) | < .001 |  | 0.067 (0.030, 0.107) | .002 |  |
| Total | 2.567 (1.776, 2.672) | < .001 |  | 0.079 (0.041, 0.122) | < .001 |  |

The models were adjusted for age, race, education, marital status, poverty status, smoking status, alcohol consumption, physical activity, HTN, DM, CVD, cancer, CKD, BMI, and TG. PhenoAge = phenotypic age, PhenoAgeAccel = phenotypic age acceleration, CI = confidence interval.


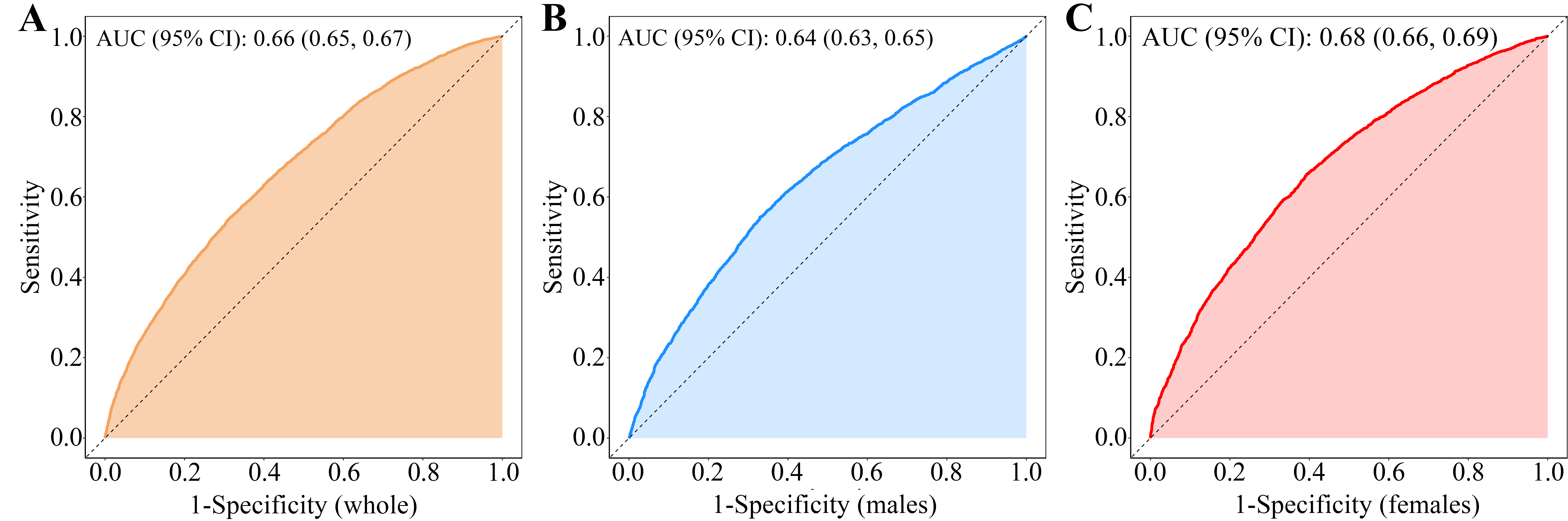


**Figure S1.** Receiver operating characteristic curves after additional adjustment for depression and sleep disorders. Fatty liver index predictive accuracy for the risk of phenotypic age acceleration in the whole population (A), males (B) and females (C). AUC = area under the curve, Cl = confidence interval.


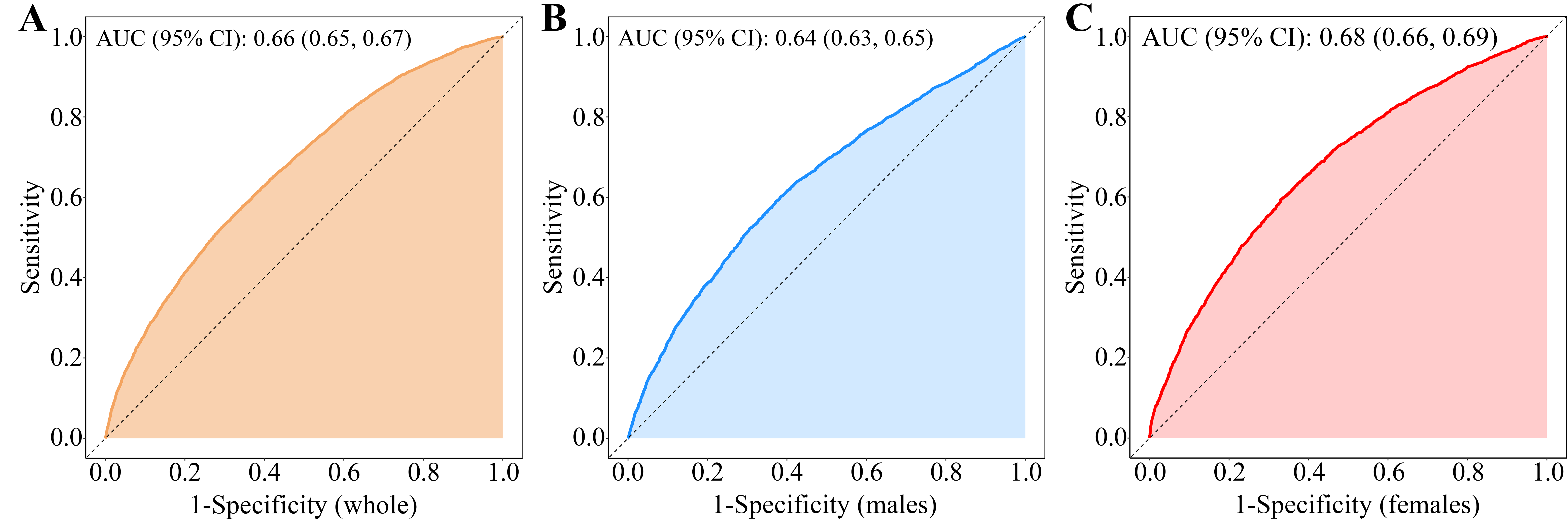


**Figure S2.** Receiver operating characteristic curves with unweighted multivariable regression. Fatty liver index predictive accuracy for the risk of phenotypic age acceleration in the whole population (A), males (B) and females (C). AUC = area under the curve, Cl = confidence interval.


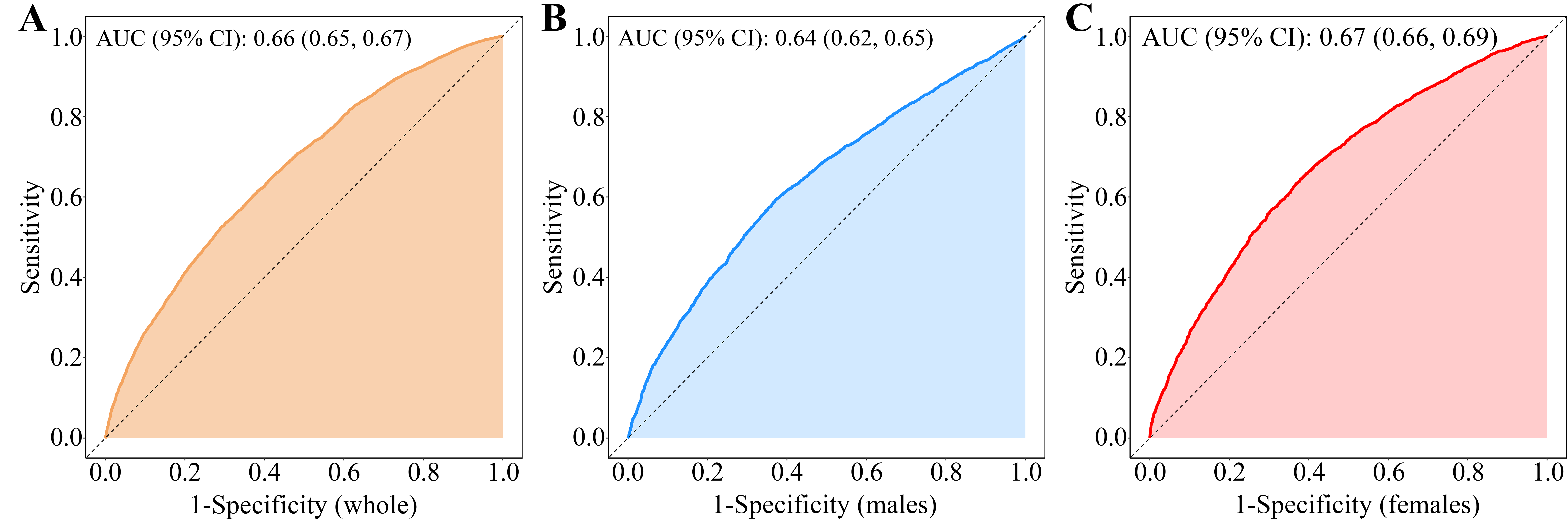


**Figure S3.** Receiver operating characteristic curves following exclusion of participants aged ≥80 years. Fatty liver index predictive accuracy for the risk of phenotypic age acceleration in the whole population (A), males (B) and females (C). AUC = area under the curve, Cl = confidence interval.


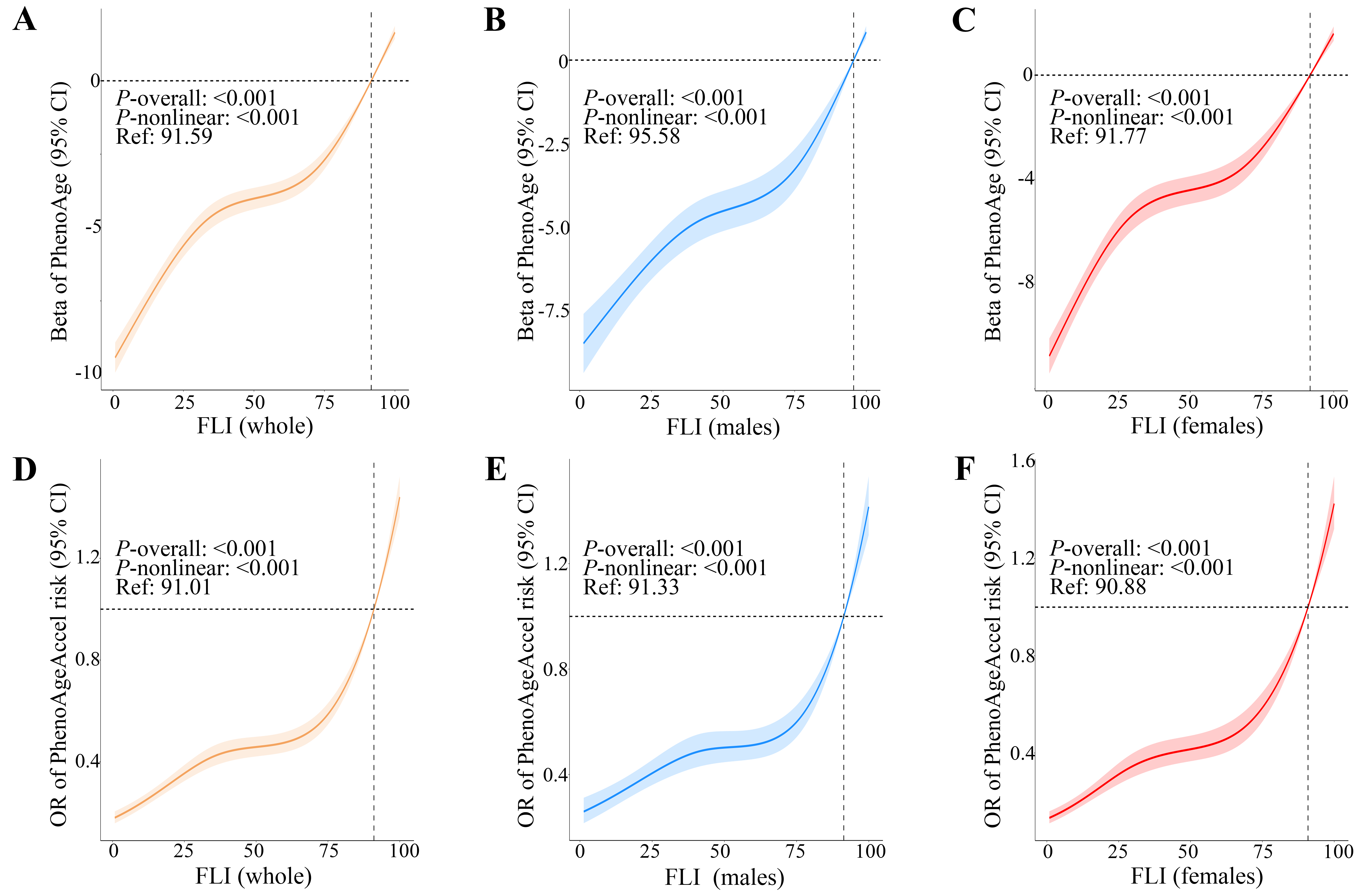


**Figure S4.** RCSs for the FLI–BA association after additional adjustment for depression and sleep disorders. Associations between FLI and PhenoAge/PhenoAgeAccel risk among the whole population (A, D), males (B, E), and females (C, F). RCS = Restricted cubic spline, BA = biological aging, Cl = confidence interval, OR = odds ratio, FLI = fatty liver index, PhenoAge = phenotypic age, PhenoAgeAccel = phenotypic age acceleration.


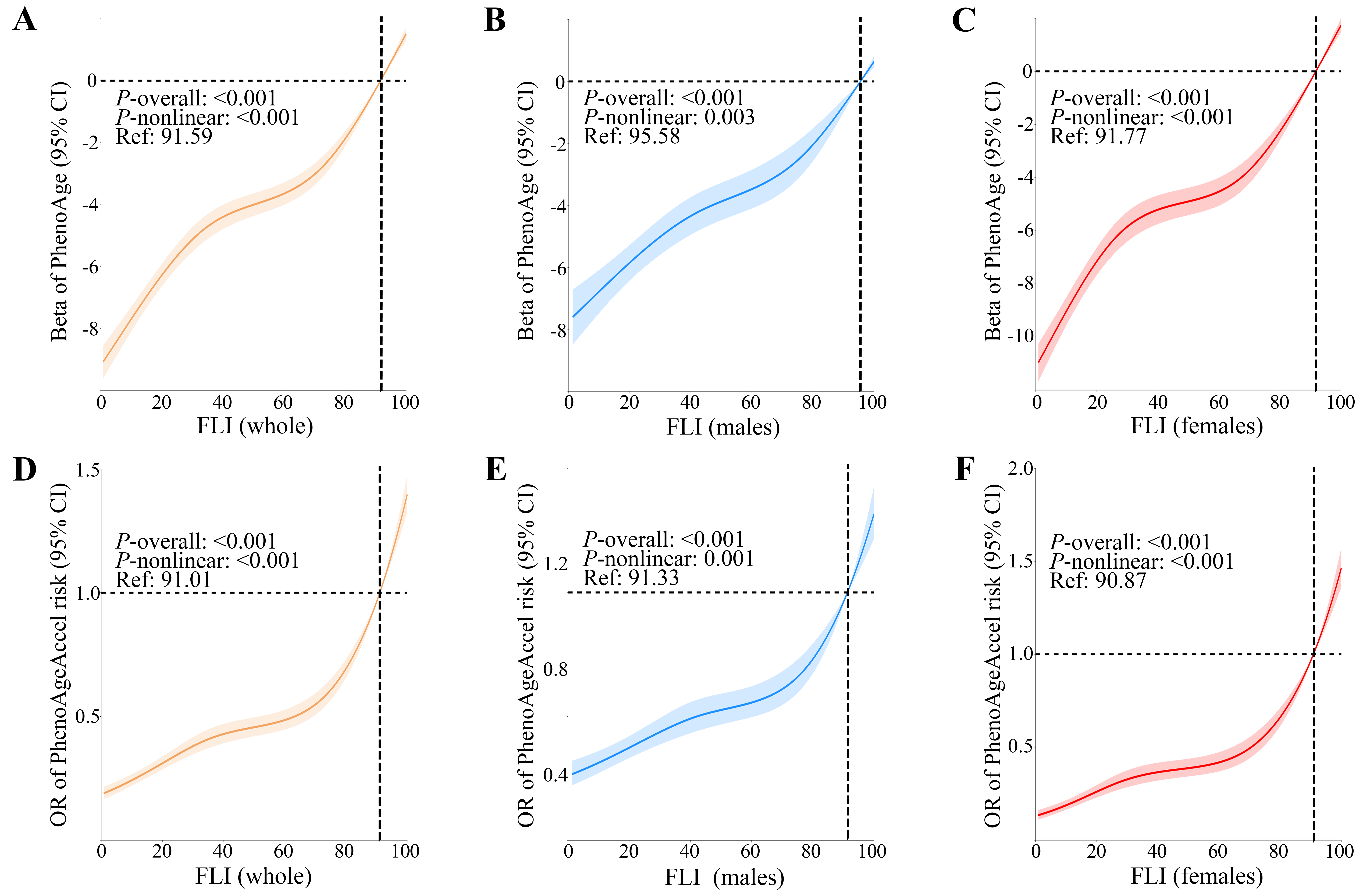


**Figure S5.** RCSs for the FLI–BA association with unweighted multivariable regression. Associations between FLI and PhenoAge/PhenoAgeAccel risk among the whole population (A, D), males (B, E), and females (C, F). RCS = Restricted cubic spline, BA = biological aging, Cl = confidence interval, OR = odds ratio, FLI = fatty liver index, PhenoAge = phenotypic age, PhenoAgeAccel = phenotypic age acceleration.


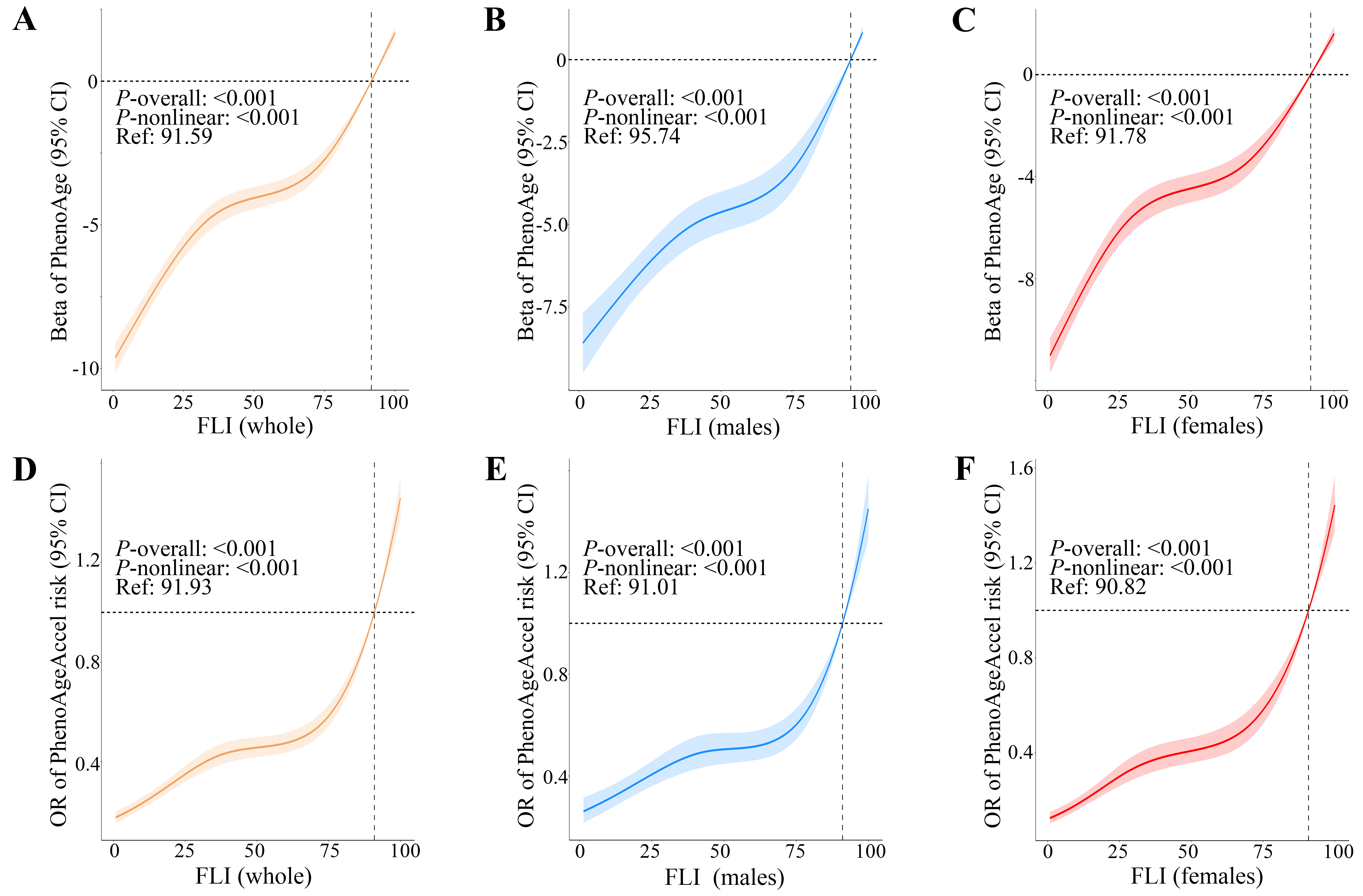


**Figure S6.** RCSs for the FLI–BA association following exclusion of participants aged ≥80 years. Associations between FLI and PhenoAge/PhenoAgeAccel risk among the whole population (A, D), males (B, E), and females (C, F). RCS = Restricted cubic spline, BA = biological aging, Cl = confidence interval, OR = odds ratio, FLI = fatty liver index, PhenoAge = phenotypic age, PhenoAgeAccel = phenotypic age acceleration.
